# Morphological symmetry-aware generalized policy network for deep reinforcement learning

**DOI:** 10.3389/frobt.2026.1816301

**Published:** 2026-05-13

**Authors:** Ryo Hakoda, Yubin Liu, Matthew Hwang, Yoshihiro Sato, Jun Takamatsu, Katsushi Ikeuchi, Takeshi Oishi

**Affiliations:** 1 Institute of Industrial Science, The University of Tokyo, Tokyo, Japan; 2 Engineering Faculty, Kyoto University of Advanced Science, Kyoto, Japan; 3 Microsoft, Redmond, WA, United States

**Keywords:** deep reinforcement learning, humanoid robots, legged locomotion, manipulation, morphological symmetry, quadruped robots

## Abstract

Exploiting the morphological symmetry of robotic systems, such as humanoid and quadruped robots, is a promising direction for improving robot learning. In deep reinforcement learning (DRL) for robot control, prior studies have leveraged such symmetry to improve learning efficiency through data augmentation, equivariant multilayer perceptrons (EMLPs), and multi-agent reinforcement learning (MARL) formulations. However, DRL training is inherently unstable, as the data distribution strongly depends on exploration, which is driven by stochasticity in the environment. To address this issue, we propose a symmetry-assisted, general-purpose DRL framework for morphologically symmetric robots that enables stable and robust learning. The framework models the environment as a symmetric Markov decision process (MDP) and constructs a full-body policy from a single-sided base policy using symmetry operators. We further propose a symmetric PPO objective with a coupled importance-sampling ratio. This objective aligns the policy optimization process with the imposed symmetry and serves as a principled alternative to MAPPO-style multi-agent formulations. Experimental results demonstrate that the proposed method outperforms existing approaches on most symmetric tasks, while still maintaining performance comparable to or better than standard PPO on asymmetric tasks, where symmetry is less directly exploitable.

## 1 Introduction

Deep reinforcement learning (DRL) has recently demonstrated remarkable capabilities in controlling robots with high-degree-of-freedom (DoF), enabling behaviors that range from agile locomotion (Zhang et al., 2024) to dexterous manipulation (Luo et al., 2025). These advances are primarily fueled by the representational capacity of deep neural networks and scalable on-policy optimization techniques (such as PPO), which enable policies to model highly nonlinear dynamics. Despite this, the large dimensionality of the state and action spaces, combined with discrete contact events and underactuated dynamics, makes the learning of such systems fundamentally challenging (Lu et al., 2023; Barakat et al., 2023). As a result, training policies for these robots remains inefficient and prone to instability, especially for robots with many joints that operate under complex contact interactions.

Although Proximal Policy Optimization (PPO) (Schulman et al., 2017) is one of the most widely used learning algorithms in robotics, it can exhibit instability when applied to the training of humanoid and quadruped robots. PPO’s clipped objective provides a simple and effective mechanism for stabilizing on-policy updates, making it a standard baseline in many robot-learning studies. However, high actuation redundancy amplifies gradient variance, allowing exploration-induced asymmetries to accumulate and often resulting in biased behaviors.

Symmetry has long played a central role in analytical mechanics and motion generation (Ghaffari et al., 2022; Dong et al., 2023; Ordonez-Apraez et al., 2023; Apraez et al., 2025). Many robotic systems, including humanoid and quadruped robots, possess morphological symmetries, with left–right reflection being particularly notable. 1n recent years, these morphological symmetries have been incorporated into DRL by formulating the environment as a symmetric Markov decision process (MDP) (Zinkevich and Balch, 2001). In this context, a natural direction for exploiting symmetry is to embed it directly into the learning problem rather than treating it as a *post hoc* regularization or data-level heuristic.

Many approaches have been proposed to improve performance and training efficiency by exploiting the symmetry of robots; yet, each comes with its own limitations and potential drawbacks. *Loss-based* methods introduce auxiliary penalties to discourage asymmetric outputs (Yu et al., 2018; Kasaei et al., 2021; Nguyen et al., 2024; Yu et al., 2024). Such soft constraints are sensitive to the choice of penalty weights and can destabilize policy updates. *Data-augmentation* methods improve sample efficiency by adding symmetric counterparts of observed state–action pairs (Lin et al., 2020; Mittal et al., 2024; Nguyen et al., 2024; Wang et al., 2025; Bao et al., 2026). However, these approaches do not guarantee that the learned policy itself satisfies the desired symmetry. *Architectural* approaches enforce symmetry at the network architecture level, either by explicitly mirroring the robot’s configuration and formulating the problem as a multi-agent RL problem (Abdolhosseini et al., 2019; Sonmez et al., 2024; Yan et al., 2024; Li et al., 2025), or by using symmetry-aware network architectures such as group-equivariant multilayer perceptrons (EMLPs) (Finzi et al., 2021; Kirsch et al., 2022; Wang et al., 2022; Liu et al., 2023; Su et al., 2024; Huang et al., 2023). Yet, these approaches do not guarantee stable policy updates against asymmetric samples that inevitably occur in random exploration in the environment.

In this paper, we present a symmetry-based DRL framework that leverages morphological symmetry at both the level of policy construction and optimization. We formulate the environment as a symmetric MDP equipped with left–right symmetry operators defined over the state and action spaces. Based on this formulation, we decompose the robot’s configuration and action spaces into left and right components and train a single base policy that produces actions for one side of the robot, in line with MARL-based formulations (Abdolhosseini et al., 2019; Yan et al., 2024). Full-body actions are obtained by applying the symmetry operators to the base policy actions produced from mirrored observations. This approach ensures that the resulting policy is equivariant with respect to the prescribed symmetry, while reducing the effective action dimensionality and maintaining a network architecture as simple as a standard MLP.

Beyond the policy architecture, we argue that the learning objective itself must respect the joint symmetry of the robot. To this end, we derive an objective that treats the left and right actions as a single joint policy update, rather than as two loosely coupled agents, as in standard MAPPO-style MARL (Yu et al., 2022). Concretely, we define a coupled importance-sampling ratio based on the full symmetric policy and apply the PPO clipping to this ratio, ensuring that both sides of the robot are updated in a coordinated, symmetry-consistent manner. This stands in contrast to MARL formulations that share parameters across sides but perform per-side clipping, which can lead to partially inconsistent updates when the collected experience is asymmetric.

Our main contributions are summarized as follows:

- We formulate a symmetry-based general-purpose DRL framework for morphologically symmetric robots. Our approach models the environment as a symmetric MDP and explicitly constructs a full-body policy from a single-sided base policy via symmetry operators.
- We derive a symmetric PPO objective based on a coupled importance-sampling ratio for the full policy. This formulation aligns the optimization procedure with the imposed symmetry and can be regarded as a principled alternative to MAPPO-style multi-agent objectives for symmetric robots.
- We conduct extensive empirical evaluation on ten symmetric and six asymmetric tasks across five robotic platforms, including real humanoid and quadruped robots, comparing PPO (Schulman et al., 2017), data augmentation (Mittal et al., 2024), EMLP (Su et al., 2024), MARL (Yan et al., 2024), and our method.

## 2 Methodology

In this section, we first present brief preliminaries on DRL, the morphological symmetry of robotic systems, and the corresponding symmetric MDP formulation, and then propose a symmetry-based DRL framework that exploits these structures in both policy construction and optimization. For clarity, Tables 1, 2 summarize the main symbols, spaces, and symmetry-related operators used throughout this section.

**TABLE 1:** Summary of the main spaces, variables, and objectives.

Symbol	Meaning
S,A,Q	State, action, and joint-configuration spaces
$Q_{\text{l}},Q_{\text{r}}$	Left and right joint-configuration spaces
$A_{\text{l}},A_{\text{r}}$	Left and right action spaces
s,a,q	State, action, and joint configuration
$q_{\text{l}},q_{\text{r}}$	Left and right components of the joint configuration
$a_{\text{l}},a_{\text{r}}$	Left and right action components
$\pi_{\theta }$	Full-body stochastic policy
${\hat{\pi }}_{\theta }$	Base policy defined on the left action space
$V_{\phi },V_{\text{sym}}$	Critic network and its symmetrized output
$\rho_{\theta }$	Standard PPO importance-sampling ratio
$\rho_{\text{l}},\rho_{\text{r}}$	Per-side importance-sampling ratios
$\rho_{\text{sym}}$	Coupled symmetric importance-sampling ratio
$A,A_{\text{l}},A_{\text{r}}$	Full and per-side advantage functions
$J_{\text{PPO}},J_{\text{MARL}},J_{\text{sym}}$	Standard PPO, MARL-style PPO, and proposed symmetric PPO objectives

**TABLE 2:** Summary of symmetry-related operators and maps.

Symbol	Domain/Codomain	Meaning
$M_{s}$	S→S	Symmetry operator on the state/observation space
$M_{a}$	A→A	Symmetry operator on the full action space
$M_{q}$	Q→Q	Symmetry operator on the full joint configuration
$M_{\text{split}}$	Bijection	Split operator on either configurations or actions: $X\to X_{\text{l}}\times X_{\text{r}}$ , where X∈{Q,A}
${\hat{M}}_{q}$	$Q_{\text{l}}\to Q_{\text{r}}$	Left-to-right configuration correspondence map
${\hat{M}}_{a}$	$A_{\text{l}}\to A_{\text{r}}$	Left-to-right action correspondence map
$P_{\text{intr}},P_{\text{extr}}$	Permutation operators	Permute intrinsic and extrinsic observation entries
$F_{\text{intr}},F_{\text{extr}}$	Value-flipping operators	Flip signs to intrinsic and extrinsic observation entries

### 2.1 Preliminary

#### 2.1.1 Problem formulation and PPO objective

In deep reinforcement learning (DRL), an agent learns optimal actions from experience obtained through interactions with the environment. The environment is modeled as a Markov decision process (MDP) (Sutton and Barto, 1998), represented as a tuple $\left(S,A,r,T,p_{0}\right),$ where S is the state space, A is the action space, and r:S×A→R is the reward function. The transition density function T:S×A×S→[0,1] specifies the transition probability, where $T\left(s^{\prime }|s,a\right)$ denotes the probability of transitioning to state $s^{\prime }$ from state s when taking action a . The initial state distribution $p_{0}:S\to \left[0,1\right]$ gives the probability that the Markov process starts in state s .

The agent interacts with the environment by selecting actions according to a stochastic policy π:S×A→[0,1] , where π(a∣s) denotes the probability of taking action a in state s . In robot control tasks, the action space is typically continuous and can be written as $A\subset R^{n}$ , where n is the dimension of the action space. In this setting, we model the policy $\pi_{\theta }$ as a Gaussian

$$\pi_{\theta }\left(a|s\right)=N\;\left(a\;;\;\mu_{\theta }\left(s\right),\sigma_{\theta }\left(s\right)\right),$$

where $\mu_{\theta }\left(s\right)\in R^{n}$ and $\sigma_{\theta }\left(s\right)\in R_{>0}^{n}$ are the mean and standard-deviation vectors, parameterized by θ .

In practice, we optimize the policy parameters θ using Proximal Policy Optimization (PPO) (Schulman et al., 2017). In PPO, the policy network is referred to as the *actor* network, and it is updated by maximizing a clipped surrogate objective based on an importance-sampling ratio $\rho_{\theta }\left(s,a\right)$ .

$$\rho_{\theta }\left(s,a\right):=\frac{\pi_{\theta }\left(a|s\right)}{\pi_{\text{old}}\left(a|s\right)},$$

where $\pi_{\text{old}}$ denotes the actor policy used to collect data. The standard PPO objective is defined with the advantage function A(s,a) , as follows:

$$J_{\text{PPO}}\left(\theta \right)=E_{\left(s,a\right)∼D}\left[\min\left(\rho_{\theta }\left(s,a\right)A\left(s,a\right),\text{clip }\left(\rho_{\theta }\left(s,a\right),1-\epsilon ,1+\epsilon \right)A\left(s,a\right)\right)\right],$$

where D is the dataset collected under $\pi_{\text{old}}$ , and ϵ>0 is a hyperparameter for the clipping function clip () that controls the update range. In practice, this surrogate is combined with a value-function loss and an entropy bonus to stabilize learning.

#### 2.1.2 Robot morphology-based symmetry groups

Many robotic systems, such as humanoid and quadruped robots, can be modeled using a left–right reflection symmetry group, denoted by $G:=C_{2}=\left\langle g|g^{2}=e\right\rangle$ , where e denotes the identity element and g is the generator representing the left–right reflection (Finzi et al., 2021). This means that their links and joints are arranged symmetrically with respect to the sagittal plane of the body. The generator g induces symmetry operators on the state and action spaces, which we denote by $M_{s}:S\to S$ and $M_{a}:A\to A$ , respectively. Since $C_{2}$ is a two-element group with $g^{2}=e$ , these operators are involutions, i.e., $M_{s}^{2}=Id$ and $M_{a}^{2}=Id$ , and hence $M_{s}^{-1}=M_{s}$ and $M_{a}^{-1}=M_{a}$ . In practice, $M_{s}$ and $M_{a}$ can be implemented as linear maps that combine permutations of coordinates with flipping operations (and, if necessary, constant offsets).

#### 2.1.3 Symmetry constraints in DRL

To incorporate symmetry into DRL, we model the environment as a symmetric MDP (Zinkevich and Balch, 2001). An MDP $\left(S,A,r,T,p_{0}\right)$ is defined as symmetric if there exist symmetry operators $M_{s}$ and $M_{a}$ under which the reward function, the transition density function, and the initial state distribution are invariant:

$$r\left(M_{s}\left(s\right),M_{a}\left(a\right)\right)=r\left(s,a\right),$$

$$T\left(M_{s}\left(s^{\prime }\right)|M_{s}\left(s\right),M_{a}\left(a\right)\right)=T\left(s^{\prime }|s,a\right),$$

$$p_{0}\left(M_{s}\left(s\right)\right)=p_{0}\left(s\right).$$

Under this symmetry assumption, the optimal policy $\pi^{*}:S\times A\to \left[0,1\right]$ and the optimal value function $V^{*}:S\to R$ are also invariant:

$$\pi^{*}\left(M_{a}\left(a\right)|M_{s}\left(s\right)\right)=\pi^{*}\left(a|s\right),$$

$$V^{*}\left(M_{s}\left(s\right)\right)=V^{*}\left(s\right).$$

In DRL for robotic control, a common way to exploit morphological symmetry is to impose invariance on the policy and value function with respect to the symmetry operators:

$$\pi \left(M_{a}\left(a\right)|M_{s}\left(s\right)\right)=\pi \left(a|s\right),$$ (1)

$$V\left(M_{s}\left(s\right)\right)=V\left(s\right)$$ (2)

### 2.2 Policy symmetrization by multi agents

We propose a DRL framework that exploits the morphological symmetry of the robot and redefines the environment as a multi-agent RL system to reduce the complexity of the action space. We define the configuration space of the robot as $Q\subset R^{\text{DoF}}$ , where DoF denotes the number of degrees of freedom of the system. For a symmetric system, we assume there exists a configuration-level symmetry operator $M_{q}:Q\to Q$ associated with the generator g of the symmetry group. As in the case of $M_{s}$ and $M_{a}$ , this operator is an involution, i.e., $M_{q}^{2}=Id$ and thus $M_{q}^{-1}=M_{q}$ .

**FIGURE 1:**
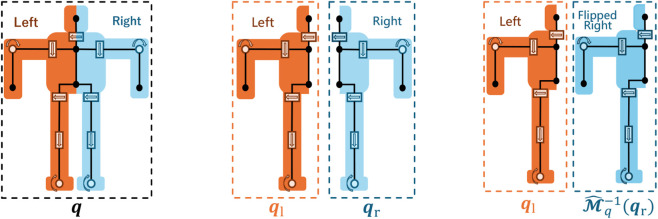
Example of configuration-space decomposition. Left: a full-body humanoid with morphological symmetry, whose joint state is denoted by q∈Q . Center: decomposition of the humanoid into left and right halves, whose joint states are represented as $q_{\text{l}}\in Q_{\text{l}}$ and $q_{\text{r}}\in Q_{\text{r}}$ , respectively. Right: construction of two equivalent single-sided systems by flipping the right half. Their joint states are $q_{\text{l}}\in Q_{\text{l}}$ and ${\hat{M}}_{\text{q}}^{-1}\left(q_{\text{r}}\right)\in Q_{\text{l}}$ , respectively. They share the same configuration space and can be controlled by the same policy $\hat{\pi }$ .

Following Abdolhosseini et al. (2019) and Yan et al. (2024), we introduce a decomposition of the joint configuration q∈Q into left and right joint states $q_{\text{l}}\in Q_{\text{l}}$ and $q_{\text{r}}\in Q_{\text{r}}$ with respect to the sagittal plane, as follows (Figure 1):

$$\left(q_{\text{l}},q_{\text{r}}\right):=M_{\text{split}}\left(q\right),$$

where the mapping $M_{\text{split}}:Q\to Q_{\text{l}}\times Q_{\text{r}}$ is a bijection between Q and the product of left and right configuration spaces $Q_{\text{l}}$ and $Q_{\text{r}}$ . Joints that lie on the sagittal plane, such as neck or waist joints, are conceptually represented as pairs of virtual left and right joints so that each physical joint is assigned to exactly one of $q_{\text{l}}$ or $q_{\text{r}}$ .

For a symmetric system, the following properties hold: (i) $\dim\left(Q_{\text{l}}\right)=\dim\left(Q_{\text{r}}\right)$ , and (ii) there exists a bijection ${\hat{M}}_{q}:Q_{\text{l}}\to Q_{\text{r}}$ such that, for all q∈Q ,

$$\left(q_{\text{l}},q_{\text{r}}\right)=M_{\text{split}}\left(q\right),$$

$$M_{q}\left(q\right)=M_{\text{split}}^{-1}\left({\hat{M}}_{q}^{-1}\left(q_{\text{r}}\right),\;{\hat{M}}_{q}\left(q_{\text{l}}\right)\right).$$ (3)

Intuitively, ${\hat{M}}_{q}$ maps a left-side configuration to the corresponding right-side configuration, and $M_{q}$ acts on the full configuration by swapping the left and right components through ${\hat{M}}_{q}$ and its inverse.

As a concrete example, consider the humanoid robot in Figure 1. For $M_{\text{split}}$ , the full-body joint vector q is divided into two joint vectors, $q_{\text{l}}$ and $q_{\text{r}}$ , based on the robot’s symmetry plane. Joints located on the symmetry plane are included in both vectors. For ${\hat{M}}_{q}$ , the left-side joint vector $q_{\text{l}}$ is reflected across the symmetry plane by flipping the signs of the joints whose rotation axes are parallel to the plane. In the robot shown in Figure 3, this includes, for example, the neck, elbow, hip, and ankle joints.

**FIGURE 2:**
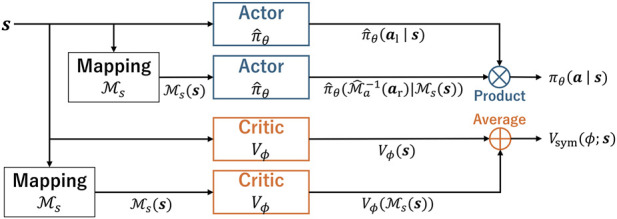
Network architecture of the proposed framework. Modules with the same color (blue or orange) share network parameters.

We now construct a policy that operates only on the left side and is mirrored to create a full-body policy. The block diagram of the policy is shown in Figure 2. In the figure, the entire policy is represented as two policies that share parameters. Let ${\hat{\pi }}_{\theta }$ denote the base policy with the left action space $A_{\text{l}}$ , with dimensions that match $Q_{\text{l}}$ . Analogously to the joint configuration, we assume that the full action space A can be decomposed as

$$M_{\text{split}}:A\to A_{\text{l}}\times A_{\text{r}},$$

where $A_{\text{l}}$ and $A_{\text{r}}$ are the left and right action spaces, respectively, and $M_{\text{split}}$ is a bijection. With a slight abuse of notation, we use the same symbols $M_{\text{split}}$ and $M_{\text{split}}^{-1}$ for the configuration and action spaces.

By reusing the construction of ${\hat{M}}_{q}$ , we introduce an action-level symmetry mapping ${\hat{M}}_{a}:A_{\text{l}}\to A_{\text{r}}$ between the left and right action spaces. Given a state s , we define the full action a∈A generated by the policy as

$$a:=M_{\text{split}}^{-1}\left(a_{\text{l}},\;a_{\text{r}}\right),$$ (4)

$$a_{\text{l}}∼{\hat{\pi }}_{\theta }\left(\cdot |s\right),$$ (5)

$$a_{\text{r}}:={\hat{M}}_{a}\left(a_{\text{r}}^{\prime }\right),\;a_{\text{r}}^{\prime }∼{\hat{\pi }}_{\theta }\left(\cdot |M_{s}\left(s\right)\right).$$ (6)

Here, $a_{\text{l}}$ is a left-side action sampled with state s , $a_{\text{r}}^{\prime }$ is a left-side action sampled with the mirrored state $M_{s}\left(s\right)$ , and ${\hat{M}}_{a}\left(a_{\text{r}}^{\prime }\right)$ yields the corresponding right-side action. Finally, $M_{\text{split}}^{-1}$ recombines the left and right components into a full action in A . Within this framework, the operation of a single robot is treated as a multi-agent task involving two agents, each controlling only one-half of the body.

As a simple example, consider a locomotion task with the robot shown in Figure 1. Let the state s consist only of the joint positions q , and let the action a be the target joint positions $\dot{q}\in Q$ . In this case, $M_{s}\left(s\right)=M_{q}\left(q\right)$ . By feeding s and $M_{s}\left(s\right)$ into the base policy ${\hat{\pi }}_{\theta }$ , we obtain the side-wise actions $a_{\text{l}}$ and $a_{\text{r}}^{\prime }$ , which are then combined in the reverse manner of the joint-splitting operation to produce the full-body action a , i.e., the target joint positions $\dot{q}$ .

We now show that the resulting full policy $\pi_{\theta }$ satisfies the invariance condition (Equation 1). Let $\left(a_{\text{l}},a_{\text{r}}\right)=M_{\text{split}}\left(a\right)$ . By construction (Equations 4–6), the joint density of a under $\pi_{\theta }$ can be written as

$$\pi_{\theta }\left(a|s\right)={\hat{\pi }}_{\theta }\left(a_{\text{l}}|s\right)\;{\hat{\pi }}_{\theta }\left({\hat{M}}_{a}^{-1}\left(a_{\text{r}}\right)|M_{s}\left(s\right)\right).$$

Analogously to Equation 3, we define the action-level symmetry operator

$$M_{a}\left(a\right):=M_{\text{split}}^{-1}\left({\hat{M}}_{a}^{-1}\left(a_{\text{r}}\right),\;{\hat{M}}_{a}\left(a_{\text{l}}\right)\right),$$

So that, if $\left({\tilde{a}}_{\text{l}},{\tilde{a}}_{\text{r}}\right)=M_{\text{split}}\left(M_{a}\left(a\right)\right)$ , we have ${\tilde{a}}_{\text{l}}={\hat{M}}_{a}^{-1}\left(a_{\text{r}}\right)$ and ${\tilde{a}}_{\text{r}}={\hat{M}}_{a}\left(a_{\text{l}}\right)$ . Applying the same construction (Equations 4–6) at the mirrored state $M_{s}\left(s\right)$ yields.

$$\pi_{\theta }\left(M_{a}\left(a\right)|M_{s}\left(s\right)\right)={\hat{\pi }}_{\theta }\left({\tilde{a}}_{\text{l}}|M_{s}\left(s\right)\right)\;{\hat{\pi }}_{\theta }\left({\hat{M}}_{a}^{-1}\left({\tilde{a}}_{\text{r}}\right)|M_{s}^{2}\left(s\right)\right)$$

$$={\hat{\pi }}_{\theta }\left({\hat{M}}_{a}^{-1}\left(a_{\text{r}}\right)|M_{s}\left(s\right)\right)\;{\hat{\pi }}_{\theta }\left(a_{\text{l}}|s\right)$$

$$=\pi_{\theta }\left(a|s\right),$$

where we used the facts that ${\hat{M}}_{a}^{-1}◦{\hat{M}}_{a}=Id$ and $M_{s}^{2}=Id$ . Therefore, the constructed policy $\pi_{\theta }$ satisfies the invariance condition

$$\pi_{\theta }\left(M_{a}\left(a\right)|M_{s}\left(s\right)\right)=\pi_{\theta }\left(a|s\right),$$

and hence is consistent with Equation 1.

### 2.3 Critic network symmetrization

Same as the standard PPO, we train the critic network $V_{\phi }:S\to R$ , which is parametrized by ϕ . To maintain invariance as denoted in Equation 2, the value function output is calculated by,

$$V_{\text{sym}}\left(\phi ;s\right)=\frac{V_{\phi }\left(s\right)+V_{\phi }\left(M_{\text{s}}\left(s\right)\right)}{2}.$$

### 2.4 Coupled objective function

In addition to the proposed symmetric policy architecture, we further modify the objective of the underlying DRL algorithm. By working in a smaller action space $A_{\text{l}}$ for the base policy (and generating right-side actions by symmetry), the complexity of the policy network can be reduced. However, the left and right action spaces are closely related because they jointly control a single robot; therefore, policy updates must be performed with caution. In Yan et al. (2024), the authors directly apply a multi-agent RL algorithm based on PPO (Schulman et al., 2017). In a simple MARL implementation (e.g., MAPPO (Yu et al., 2022)), the actor objective is given by

$$\begin{array}{ll} J_{\text{MARL}}\left(\theta \right) & =E_{\left(s,a_{\text{l}},a_{\text{r}}\right)∼D}\\ & \;\left[\frac{1}{2}\underset{i\in \left\{l,r\right\}}{\sum }\min\left(\rho_{i}\left(\theta ;s,a_{i}\right)\;A_{i}\left(s,a_{i}\right),\text{clip }\left(\rho_{i}\left(\theta ;s,a_{i}\right),1-\epsilon ,1+\epsilon \right)\;A_{i}\left(s,a_{i}\right)\right)\right]. \end{array}$$

The importance-sampling ratios for the left and right sides are defined as.

$$\rho_{\text{l}}\left(\theta ;s,a_{\text{l}}\right)=\frac{{\hat{\pi }}_{\theta }\left(a_{\text{l}}|s\right)}{{\hat{\pi }}_{\text{old}}\left(a_{\text{l}}|s\right)},$$

$$\rho_{\text{r}}\left(\theta ;s,a_{\text{r}}\right)=\frac{{\hat{\pi }}_{\theta }\left({\hat{M}}_{\text{a}}^{-1}\left(a_{\text{r}}\right)|M_{s}\left(s\right)\right)}{{\hat{\pi }}_{\text{old}}\left({\hat{M}}_{\text{a}}^{-1}\left(a_{\text{r}}\right)|M_{s}\left(s\right)\right)},$$

where ${\hat{\pi }}_{\text{old}}$ denotes the policy before the update, D denotes the data distribution collected by ${\hat{\pi }}_{\text{old}}$ , and $A_{i}\left(s,a\right)$ is the advantage function computed using the GAE estimator (Schulman et al., 2016).

However, this objective treats the left and right action components as if they were controlled by two independent agents that share parameters but are updated using separate importance-sampling ratios. Since the full policy $\pi_{\theta }$ is constructed as the left policy with inputs of the original state and the mirrored state, this decoupled treatment can lead to inconsistent updates between the two sides, even though they jointly control a single robot. In particular, the clipping is applied independently to $\rho_{\text{l}}$ and $\rho_{\text{r}}$ , which may over- or under-penalize updates for configurations where the symmetry plays an essential role.

To better respect the symmetric structure of the policy, we consider the full policy

$$\pi_{\theta }\left(a|s\right)={\hat{\pi }}_{\theta }\left(a_{\text{l}}|s\right)\;{\hat{\pi }}_{\theta }\left({\hat{M}}_{\text{a}}^{-1}\left(a_{\text{r}}\right)|M_{s}\left(s\right)\right),$$

and define a single coupled importance-sampling ratio

$$\rho_{\text{sym}}\left(\theta ;s,a\right):=\frac{\pi_{\theta }\left(a|s\right)}{\pi_{\text{old}}\left(a|s\right)}=\rho_{\text{l}}\left(\theta ;s,a_{\text{l}}\right)\;\rho_{\text{r}}\left(\theta ;s,a_{\text{r}}\right),$$

where $\pi_{\text{old}}$ is defined similarly using ${\hat{\pi }}_{\text{old}}$ . Since our method assumes a symmetric MDP environment with reward and value function symmetry, the advantage function is invariant and can be defined as $A\left(s,a\right)=A_{\text{l}}\left(s,a_{\text{l}}\right)=A_{\text{r}}\left(s,a_{\text{r}}\right)$ . We then replace the MAPPO-style objective by the following symmetric PPO objective:

$$J_{\text{sym}}\left(\theta \right)=E_{\left(s,a\right)∼D}\left[\min\left(\rho_{\text{sym}}\left(\theta ;s,a\right)\;A\left(s,a\right),\text{clip }\left(\rho_{\text{sym}}\left(\theta ;s,a\right),1-\epsilon ,1+\epsilon \right)\;A\left(s,a\right)\right)\right].$$ (7)

This objective performs clipping based on the ratio of the *coupled* symmetric policy rather than on the per-side ratios separately, and thus updates the left and right actions in a joint manner.

By definition, the symmetric ratio is invariant under the symmetry operators: for any (s,a) ,

$$\rho_{\text{sym}}\left(\theta ;M_{s}\left(s\right),M_{a}\left(a\right)\right)=\rho_{\text{sym}}\left(\theta ;s,a\right),$$

because $\pi_{\theta }$ itself satisfies $\pi_{\theta }\left(M_{a}\left(a\right)|M_{s}\left(s\right)\right)=\pi_{\theta }\left(a|s\right)$ . Consequently, the coupled PPO objective (Equation 7) is consistent with the invariance condition (Equation 1) and encourages policy updates that preserve the underlying morphological symmetry of the robot.

### 2.5 Observation symmetrization

#### 2.5.1 Symmetric environment

We now describe how we define the symmetry operator on observations, $M_{s}:S\to S$ , for all states s∈S . In DRL for robotic control, the observation is typically composed of *intrinsic* and *extrinsic* components. The intrinsic component contains proprioceptive information of the robot, such as joint and link states. The extrinsic component encodes information of the external environment, such as the states of manipulated objects. For locomotion tasks, most of the observations are intrinsic, whereas manipulation tasks require rich extrinsic information.

We represent the observation as a tuple

$$s:=\left(s_{\text{intr}},s_{\text{extr}},1,0\right),$$

where $s_{\text{intr}}$ and $s_{\text{extr}}$ denote the intrinsic and extrinsic components, respectively. As described above, $M_{s}$ is implemented as a combination of permutation and value-flipping (with constant offsets) operators. Let P denote a permutation operator and F a value-flipping operator. We then define the symmetrized observation as

$$M_{s}\left(s\right)=\left(F_{\text{intr}}◦P_{\text{intr}}\left(s_{\text{intr}}\right),\;F_{\text{extr}}◦P_{\text{extr}}\left(s_{\text{extr}}\right),\;0,1\right),$$ (8)

where $P_{\text{intr}}$ and $P_{\text{extr}}$ are the permutation operators for intrinsic and extrinsic components, and $F_{\text{intr}}$ and $F_{\text{extr}}$ are the corresponding value-flipping operators.

The last two entries form a one-hot vector that is flipped under $M_{s}$ , i.e., (1,0)↦(0,1) . This prevents the observation from becoming *neutral* in the sense that we avoid configurations for which $M_{s}\left(s\right)=s$ and, consequently, $M_{a}\left(a\right)=a$ . Existing work on locomotion, such as Abdolhosseini et al. (2019); Mittal et al. (2024), uses a phase signal Liu et al. (2016) to characterize the gait cycle of each leg and to avoid such neutral configurations. However, phase signals rely on the periodicity of the motion, whereas our method targets more general tasks, including manipulation, which do not necessarily exhibit periodic behavior. We therefore use the one-hot indicator instead of a phase signal.

#### 2.5.2 Asymmetric environment

Although the proposed method assumes that the underlying environment is symmetric, it is rare in practice for the dataset collected by an agent to be perfectly symmetric. In this setting, we cannot directly use the observation symmetry operator $M_{s}$ defined in Equation 8, because, for asymmetric tasks, there is no well-defined permutation $P_{\text{extr}}$ on the extrinsic states that pairs objects on the left with corresponding objects on the right. Instead, we introduce a modified (asymmetric) version of the operator, which we denote by the same symbol when the context is clear:

$$M_{s}\left(s\right)=\left(F_{\text{intr}}◦P_{\text{intr}}\left(s_{\text{intr}}\right),\;F_{\text{extr}}\left(s_{\text{extr}}\right),\;0,1\right).$$ (9)

This operator transforms the intrinsic state in the same way as in the symmetric case (permutation plus value flipping), while for the extrinsic state it only flips values without permuting object identities. A permutation operator for the intrinsic state can be defined from the robot’s morphological symmetry, but, in general, no such canonical permutation exists for the extrinsic state. For instance, in manipulation tasks involving multiple non-identical objects, permuting the entries of the extrinsic observation would distort the semantic correspondence among the objects. Therefore, for the extrinsic component we apply only value flipping, which mirrors the object poses while preserving object identities. As a result, $M_{s}$ is no longer an exact symmetry of the MDP, but it still provides a meaningful way to construct asymmetric counterparts of observed states.

## 3 Experimental setup

We perform experimental validation on several tasks in simulation and deploy the learned policies to real-world robots through sim-to-real transfer.

### 3.1 Comparative methods and training environments

We compared the five methods as follows: PPO, Augmentation, Equivariant MLP, Multi-Agent, and Ours. Implementation is based on the PPO algorithm from RL-Games (Makoviichuk and Makoviychuk, 2021).

- PPO: The standard PPO policy without symmetric constraints.
- Augmentation (Aug): We augment the collected sample using symmetric operations $M_{s}$ and $M_{a}$ , and append it to the dataset. We referred to Mittal et al. (2024) for implementation.
- Equivariant MLP (EMLP): We explicitly constrain the MLP network of both the policy and the critic to be equivariant to the prescribed symmetry. Concretely, EMLP represents the weights in each hidden layer as a linear combination of basis matrices that are equivariant under the symmetry transformation and treats the corresponding coefficients as trainable parameters. Our implementation follows Su et al. (2024).
- Multi-Agent (MARL): We control the robot using two agents that share the same policy network in a multi-agent reinforcement learning manner. Implementation is described in Section 2.2 and 2.3.
- Multi-Agent with Coupled loss (Ours): Our proposed method with the coupled objective is detailed in Section 2.4.

We trained all the agents in environments in the Isaac Gym simulator (Makoviychuk et al., 2021). For each task, we conducted five trials with different seed values.

### 3.2 Robotic platforms and tasks

**FIGURE 3:**
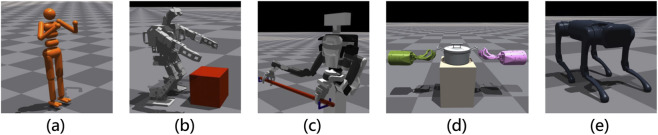
The robots used in experiments: **(a)** CG Humanoid, **(b)** Full-Body Humanoid Robot, **(c)** Upper-Body Humanoid Robot, **(d)** Bi-DexHands, and **(e)** Quadruped Robot.

We use five morphologically symmetric robotic platforms: a simulated (CG) full-body humanoid, a real full-body humanoid robot, an upper-body humanoid robot, a pair of five-fingered robotic hands, and a quadruped robot. Figure 3 shows the robots used in the experiments. Implementation details are provided in the Supplementary Material.

- CG Humanoid

For the CG humanoid, we employ the humanoid environment from IsaacGymEnvs.^1^

– Locomotion: The humanoid runs under torque control in a fixed direction.
– Backflip: The humanoid performs a backflip in place.

- Full-Body Humanoid Robot

We use a ROBOTIS OP3 full-body humanoid robot with a height of 0.5 m. All joints are driven by DYNAMIXEL servomotors controlled in position mode through the DYNAMIXEL Python API.^2^

– Locomotion: The robot walks in a commanded direction. The direction command is resampled at random time intervals.
– Pickup Box: The robot picks up a box placed 0.5–1.0 m in front of the robot. The box is a cube with an edge length of 0.15 m.

- Upper-Body Humanoid Robot

We use an upper-body humanoid robot, HIRO (KAWADA Robotics), equipped with two 1-DoF end-effectors (ROBOTIS Hand RH-P12-RN).

– Grasp Rod: The robot grasps a 1 m rod placed on a holder with both hands.

- Bi-DexHands Benchmark

We employ the Bi-DexHands benchmark (Chen et al., 2024), which contains various tasks conducted by a pair of robot dexterous hands with five fingers. We modify the environments to enforce a symmetric configuration. More specifically, since the original setup consists of two right-sided hands, we mirror the MJCF and mesh to create a left-sided hand. From the benchmark, we chose the following tasks which can be formulated as symmetric MDPs.

– Door Open Inward: The hands open double-hinged doors that can only be pulled inward.
– Lift Underarm: The hands grasp a pot by its handle with both hands and lift it upward.
– Two Catch Underarm: Each hand initially holds a ball, and the hands swap the balls by throwing them to the opposite side.

- Quadruped Robot

The quadruped robot we use is a Unitree A1.

– Locomotion: The locomotion direction is specified by linear and angular velocity commands.
– Handstand: The robot performs a handstand by balancing on its forelegs in place.

### 3.3 Asymmetric tasks

Additionally, we evaluate our method on asymmetric tasks, which do not satisfy the symmetric MDP assumption defined in Section 2.1.3. In these experiments, we investigate how asymmetric data skew affects performance by training a symmetric policy for asymmetric environments. It should be noted that we do not evaluate the augmentation-based method (Aug), since the policy network is asymmetric (as in standard PPO) and the symmetry-augmented samples are not compatible with the true environment dynamics.

For the asymmetric experiments, we use the environments from the Bi-DexHands benchmark (Chen et al., 2024).

- Block Stack: The hands stack two blocks on a table to form a tower. The desired stacking order is specified in the reward function.
- Grasp and Place: The hands place a small cube into a cup on the table. The left hand is closer to the cup, and the right hand is closer to the box.
- Scissors: The hands open a pair of scissors on the table.
- Pen: The hands remove the cap from a pen on the table. The left hand is closer to the cap, and the right hand is closer to the pen.
- Catch Underarm: The left hand throws a ball to the right hand, which catches it. This task is a one-handed version of Two Catch Underarm.
- Catch Abreast: Similar to Catch, except that the hands are positioned in parallel.

### 3.4 Evaluation metrics

We evaluated the methods with two evaluation metrics: *highest reward* and *steps-to-threshold*.

- Highest Reward: The maximum episode return during training. We trained five agents for each condition and evaluated the performance using the mean value. The range of returns among tasks differs widely, so the value is normalized by the value of the baseline PPO.
- Steps-to-Threshold: The number of training iterations required for the agent to achieve a given reward threshold. The threshold is set to 50%, 75%, 90%, and 100% of the highest reward achieved by the baseline PPO. The iteration count is normalized by the maximum number of training iterations for each task.

## 4 Results

### 4.1 Evaluation on symmetric tasks

**FIGURE 4:**
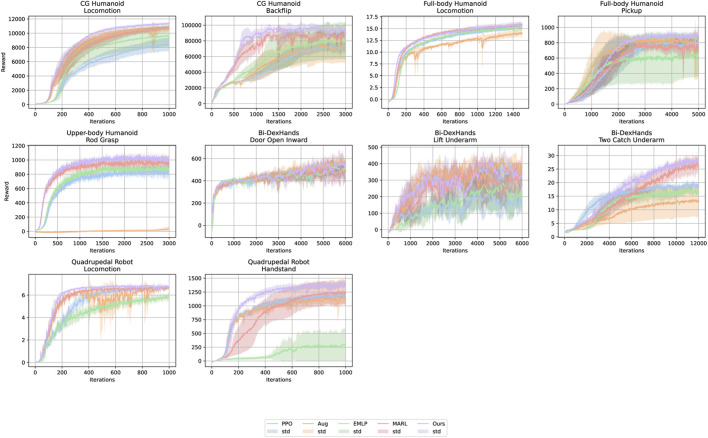
Reward curves for symmetric tasks across five runs with different random seeds. The light-colored areas indicate 95% confidence intervals.

First, we evaluate the performance of the symmetric-aware policies for the symmetric tasks. Figure 4 shows the reward curves for symmetric tasks averaged over five trials for each condition.

#### 4.1.1 Highest reward comparison

**FIGURE 5:**
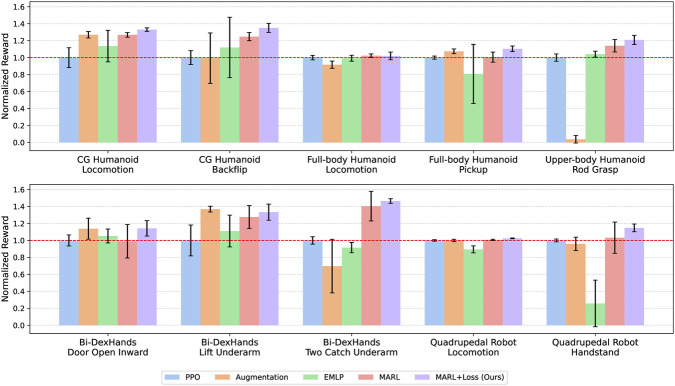
Highest reward comparison for symmetric tasks. The values are normalized by the value of PPO. The error bars indicate 95% confidence intervals.

We evaluate the reward acquisition performance of five methods: PPO, Aug, EMLP, MARL, and Ours. Figure 5 and the left column of Table 3 report the highest returns averaged over five trials for each condition; Figure 5 shows 95% confidence intervals, whereas Table 3 reports the results as mean ± standard deviation. For nine out of the ten tasks, except for the locomotion task of the full-body humanoid robot, the proposed method achieves the highest normalized reward among all methods. The augmentation-based method (Aug) and the equivariant MLP-based method (EMLP) sometimes outperform PPO, but they suffer from substantial performance drops on specific tasks (e.g., *Grasp Rod* by the upper-body humanoid robot for Aug, and *Handstand* with the quadruped robot for EMLP). The multi-agent method (MARL), which shares the same network architecture as the proposed method but uses a different objective function, consistently improves over PPO similar to Aug and EMLP, but without such pronounced degradation on particular tasks.

**TABLE 3:** Highest reward and steps-to-threshold comparison for symmetric tasks across five runs with different random seeds (mean ± std). The best value is shown in red, and the second-best value is shown in blue.

Task	Method	Highest reward (↑)	Steps-to-Threshold (↓)			
			50%	75%	90%	100%
CG humanoid locomotion	PPO	1 ± 0.123	0.276 ± 0.036	0.517 ± 0.124	0.737 ± 0.191	0.864 ± 0.142
	Aug	**1.27** ± **0.0402**	0.201 ± 0.0466	**0.255** ± **0.0524**	**0.319** ± **0.0688**	**0.385** ± **0.0942**
	EMLP	1.136 ± 0.195	0.231 ± 0.059	0.351 ± 0.201	0.484 ± 0.294	0.577 ± 0.261
	MARL	1.268 ± 0.0291	**0.187** ± **0.0331**	0.27 ± 0.0723	0.358 ± 0.117	0.423 ± 0.131
	Ours	**1.331** ± **0.0218**	**0.156** ± **0.022**	**0.201** ± **0.0221**	**0.257** ± **0.0156**	**0.311** ± **0.0191**
CG humanoid backflip	PPO	1 ± 0.0858	0.386 ± 0.0839	0.55 ± 0.0919	0.691 ± 0.181	0.817 ± 0.131
	Aug	0.993 ± 0.312	0.455 ± 0.312	0.535 ± 0.275	0.571 ± 0.256	0.671 ± 0.193
	EMLP	1.12 ± 0.374	0.392 ± 0.345	0.528 ± 0.286	0.574 ± 0.263	0.609 ± 0.255
	MARL	**1.248** ± **0.0511**	**0.133** ± **0.0251**	**0.224** ± **0.0581**	**0.265** ± **0.0723**	**0.284** ± **0.0788**
	Ours	**1.351** ± **0.0564**	**0.125** ± **0.0093**	**0.19** ± **0.01**	**0.222** ± **0.0386**	**0.235** ± **0.0458**
Full-body humanoid locomotion	PPO	1 ± 0.0278	0.0881 ± 0.0044	0.233 ± 0.033	0.58 ± 0.0706	0.943 ± 0.075
	Aug	0.917 ± 0.0446	0.101 ± 0.0044	0.387 ± 0.0195	0.905 ± 0.126	1 ± 0
	EMLP	0.993 ± 0.037	0.105 ± 0.0087	0.226 ± 0.0202	0.525 ± 0.0735	0.943 ± 0.0817
	MARL	**1.023** ± **0.0214**	**0.0824** ± **0.0084**	**0.168** ± **0.0188**	**0.379** ± **0.0459**	**0.825** ± **0.159**
	Ours	**1.02** ± **0.0479**	**0.0661** ± **0.0039**	**0.159** ± **0.0023**	**0.419** ± **0.0821**	**0.807** ± **0.193**
Full-body humanoid pickup box	PPO	1 ± 0.0196	0.337 ± 0.106	0.401 ± 0.114	0.503 ± 0.119	0.903 ± 0.159
	Aug	**1.074** ± **0.0297**	**0.227** ± **0.123**	**0.256** ± **0.12**	**0.299** ± **0.106**	**0.443** ± **0.151**
	EMLP	0.808 ± 0.365	0.368 ± 0.355	0.557 ± 0.407	0.603 ± 0.373	0.753 ± 0.34
	MARL	1.006 ± 0.0627	0.301 ± 0.0848	0.397 ± 0.112	0.489 ± 0.0924	0.847 ± 0.22
	Ours	**1.104** ± **0.0344**	**0.289** ± **0.076**	**0.325** ± **0.0834**	**0.367** ± **0.11**	**0.454** ± **0.13**
Upper-body humanoid grasp rod	PPO	1 ± 0.0454	0.129 ± 0.0122	0.226 ± 0.0175	0.434 ± 0.0845	0.872 ± 0.128
	Aug	0.0373 ± 0.0465	1 ± 0	1 ± 0	1 ± 0	1 ± 0
	EMLP	1.041 ± 0.0368	0.118 ± 0.0088	0.21 ± 0.0367	0.293 ± 0.0576	0.646 ± 0.231
	MARL	**1.14** ± **0.0772**	**0.0637** ± **0.005**	**0.106** ± **0.0203**	**0.161** ± **0.0263**	**0.263** ± **0.124**
	Ours	**1.209** ± **0.0564**	**0.0636** ± **0.0027**	**0.0989** ± **0.0089**	**0.143** ± **0.0099**	**0.211** ± **0.0252**
Bi-dexhands door open inward	PPO	1 ± 0.0686	0.0178 ± 0.0054	0.321 ± 0.147	0.66 ± 0.274	0.957 ± 0.0716
	Aug	**1.138** ± **0.13**	0.0284 ± 0.0081	0.172 ± 0.0282	**0.565** ± **0.261**	**0.645** ± **0.222**
	EMLP	1.053 ± 0.0865	0.0252 ± 0.0035	**0.132** ± **0.0601**	0.571 ± 0.225	0.828 ± 0.156
	MARL	0.99 ± 0.207	**0.0149** ± **0.0016**	0.28 ± 0.226	0.683 ± 0.312	0.713 ± 0.294
	Ours	**1.143** ± **0.0954**	**0.0175** ± **0.0065**	**0.145** ± **0.0647**	**0.49** ± **0.0796**	**0.672** ± **0.108**
Bi-dexhands lift underarm	PPO	1 ± 0.252	0.151 ± 0.0488	0.451 ± 0.312	0.708 ± 0.277	0.8 ± 0.28
	Aug	**1.423** ± **0.0558**	0.131 ± 0.0808	**0.146** ± **0.0861**	**0.172** ± **0.0954**	**0.228** ± **0.126**
	EMLP	1.269 ± 0.0855	0.238 ± 0.0506	0.362 ± 0.0347	0.484 ± 0.133	0.537 ± 0.143
	MARL	1.402 ± 0.0883	**0.0871** ± **0.007**	0.178 ± 0.0826	**0.189** ± **0.0887**	**0.199** ± **0.0867**
	Ours	**1.463** ± **0.025**	**0.114** ± **0.0552**	**0.162** ± **0.0536**	0.215 ± 0.0699	0.276 ± 0.0347
Bi-DexHands two catch underarm	PPO	1 ± 0.0455	**0.18** ± **0.0442**	**0.306** ± **0.0823**	**0.558** ± **0.0932**	0.933 ± 0.0986
	Aug	0.697 ± 0.331	0.598 ± 0.282	0.788 ± 0.291	0.86 ± 0.195	0.987 ± 0.03
	EMLP	0.915 ± 0.0638	0.286 ± 0.0565	0.521 ± 0.216	0.844 ± 0.214	1 ± 0
	MARL	**1.405** ± **0.183**	0.309 ± 0.0719	0.443 ± 0.108	0.559 ± 0.141	**0.638** ± **0.149**
	Ours	**1.465** ± **0.0283**	**0.255** ± **0.0265**	**0.345** ± **0.0467**	**0.416** ± **0.0676**	**0.478** ± **0.0679**
Quadruped robot locomotion	PPO	1 ± 0.0093	0.172 ± 0.0129	0.264 ± 0.0387	0.405 ± 0.0512	0.947 ± 0.0738
	Aug	1 ± 0.0149	0.114 ± 0.0079	**0.159** ± **0.0066**	0.245 ± 0.0307	0.932 ± 0.0858
	EMLP	0.895 ± 0.0435	0.195 ± 0.0555	0.451 ± 0.157	0.92 ± 0.115	1 ± 0
	MARL	**1.007** ± **0.0076**	**0.112** ± **0.0101**	0.159 ± 0.0093	**0.235** ± **0.0146**	**0.812** ± **0.18**
	Ours	**1.025** ± **0.0048**	**0.105** ± **0.0078**	**0.14** ± **0.0145**	**0.188** ± **0.0118**	**0.415** ± **0.047**
Quadruped robot handstand	PPO	1 ± 0.0178	**0.139** ± **0.0101**	**0.268** ± **0.0397**	**0.471** ± **0.0571**	0.943 ± 0.0733
	Aug	0.96 ± 0.0825	0.148 ± 0.0046	0.291 ± 0.0705	0.743 ± 0.265	0.946 ± 0.0825
	EMLP	0.257 ± 0.288	0.873 ± 0.19	1 ± 0	1 ± 0	1 ± 0
	MARL	**1.032** ± **0.194**	0.281 ± 0.0929	0.518 ± 0.308	0.6 ± 0.283	**0.698** ± **0.28**
	Ours	**1.148** ± **0.048**	**0.145** ± **0.0229**	**0.192** ± **0.0174**	**0.273** ± **0.0383**	**0.393** ± **0.116**

Another distinctive feature of the proposed method is its lower variance across training runs with different random seeds. This is evident from the standard deviations reported in Table 3, which summarizes the results as mean ± standard deviation Ours achieves the smallest standard deviation in 5 out of 10 tasks, and its standard deviation remains below 0.1 in all tasks.

In other words, the performance of these baselines depends strongly on stochastic factors such as network initialization and random state transitions, whereas our method is comparatively less sensitive to such sources of randomness. Collectively, these results indicate that the proposed framework is the most robust to the inevitable stochasticity in DRL among the methods considered.

#### 4.1.2 Steps-to-threshold comparison

**FIGURE 6:**
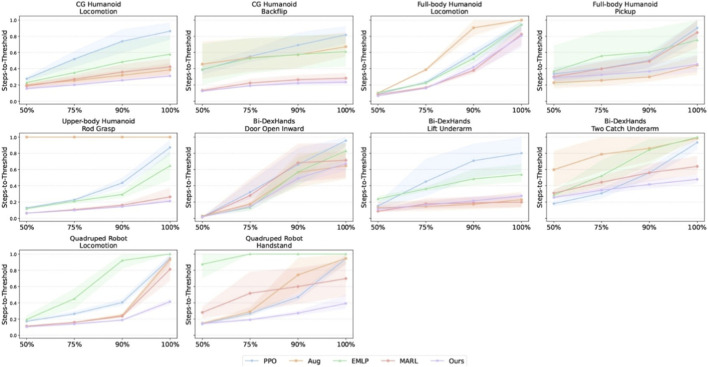
Steps-to-Threshold for symmetric tasks across five runs with different random seeds. Lower values indicate faster reward acquisition. The error bars indicate 95% confidence intervals.

Figure 6 and the right four columns of Table 3 depict the steps-to-threshold for the symmetric tasks; Figure 6 shows 95% confidence intervals, whereas Table 3 reports the results as mean ± standard deviation. Our method (Ours) reaches 100% of the PPO return fastest on seven out of ten tasks. If we also include the second-best method, Ours ranks at best or second-best on nine out of ten tasks.

PPO generally acquires rewards more slowly than the symmetry-based methods. However, in some tasks, such as *Two Catch Underarm* with Bi-DexHands and *Handstand* with the quadruped robot, PPO exhibits a characteristic pattern: it is initially the fastest method, but its learning slows down, and it is eventually overtaken by the symmetry-based approaches. By inspecting the learned behaviors, we found that this pattern arises because PPO converges to a suboptimal strategy in which only one of the two objects is transferred, yielding rapid initial reward gains but preventing further improvement toward the solution where both objects are swapped.

Aug shows highly task-dependent, “peaky” performance. It achieves reward acquisition speeds comparable to Ours on *Locomotion* with the CG humanoid, *Pickup Box* with the full-body humanoid, and *Door Open Inward* and *Lift Underarm* with Bi-DexHands, but lags behind on the other tasks.

EMLP consistently struggles to match the other methods: it never ranks in the top two on any given task.

MARL generally learns faster than PPO but falls short of Ours on most tasks. The only exception is *Lift Underarm* with Bi-DexHands, where MARL reaches the target reward faster than Ours, although its highest reward remains lower.

### 4.2 Evaluation on asymmetric tasks

**FIGURE 7:**
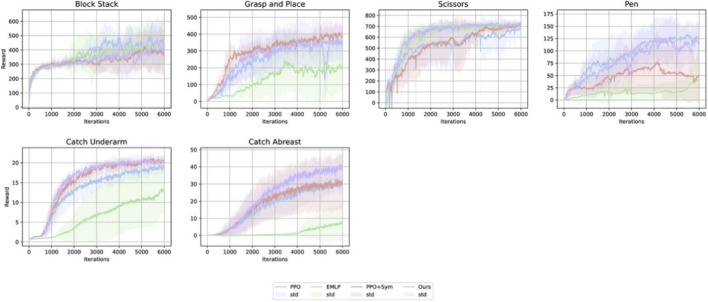
Reward curves for asymmetric tasks across five runs with different random seeds. The light-colored areas indicate 95% confidence intervals.

Next, we evaluate the performance of the symmetry-aware methods for the asymmetric tasks. We compare four methods–PPO, EMLP, MARL, and Ours–excluding Aug, since augmented data is not compatible with the asymmetric tasks. Figure 7 shows the reward curves for the asymmetric tasks averaged over five trials for each condition.

#### 4.2.1 Highest reward comparison

**FIGURE 8:**
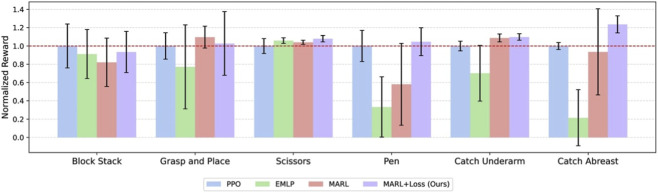
Highest reward comparison for asymmetric tasks across five runs with different random seeds. The values are normalized by the value of PPO. The error bars indicate 95% confidence intervals.

Figure 8 and the left column of Table 4 report the highest returns averaged over five trials for each condition; Figure 8 shows 95% confidence intervals, whereas Table 4 reports the results as mean ± standard deviation. Our method outperforms the other baselines on four out of the six tasks; it underperforms PPO on *Block Stack* and underperforms MARL on *Grasp and Place*. In contrast, EMLP achieves lower rewards than PPO on five out of the six tasks and, in particular, exhibits a significant performance drop on *Pen* and *Catch Abreast*. MARL attains higher returns than PPO on some tasks, but its performance shows high variance across different random seeds.

**TABLE 4:** Training speed and highest reward comparison for asymmetric tasks across five runs with different random seeds (mean ± std). The best value is shown in red, and the second-best value is shown in blue.

Task	Method	Highest reward (↑)	Steps-to-Threshold (↓)			
			50%	75%	90%	100%
Block stack	PPO	**1** ± **0.252**	**0.0849** ± **0.0177**	**0.542** ± **0.271**	**0.608** ± **0.23**	**0.63** ± **0.219**
	EMLP	0.911 ± 0.281	0.11 ± 0.0267	0.727 ± 0.36	**0.833** ± **0.3**	**0.843** ± **0.281**
	MARL	0.821 ± 0.278	**0.109** ± **0.0636**	0.829 ± 0.234	0.854 ± 0.203	0.922 ± 0.112
	Ours	**0.934** ± **0.236**	0.115 ± 0.0447	**0.673** ± **0.204**	0.858 ± 0.186	0.891 ± 0.197
Grasp and place	PPO	1 ± 0.151	**0.233** ± **0.151**	**0.347** ± **0.158**	**0.514** ± **0.274**	0.894 ± 0.237
	EMLP	0.772 ± 0.481	0.652 ± 0.329	0.684 ± 0.294	0.822 ± 0.252	0.897 ± 0.23
	MARL	**1.097** ± **0.125**	**0.145** ± **0.0314**	**0.2** ± **0.0595**	**0.33** ± **0.142**	**0.71** ± **0.308**
	Ours	**1.028** ± **0.365**	0.336 ± 0.372	0.385 ± 0.347	0.569 ± 0.397	**0.622** ± **0.368**
Scissors	PPO	1 ± 0.0855	0.176 ± 0.227	0.223 ± 0.209	0.397 ± 0.345	0.742 ± 0.252
	EMLP	**1.059** ± **0.0323**	**0.0907** ± **0.024**	**0.157** ± **0.0784**	**0.189** ± **0.0668**	**0.38** ± **0.116**
	MARL	1.041 ± 0.0237	0.242 ± 0.218	0.301 ± 0.221	0.369 ± 0.235	0.632 ± 0.303
	Ours	**1.08** ± **0.0364**	**0.0698** ± **0.0177**	**0.123** ± **0.0137**	**0.187** ± **0.047**	**0.384** ± **0.207**
Pen	PPO	**1** ± **0.178**	**0.257** ± **0.17**	**0.511** ± **0.327**	**0.562** ± **0.312**	**0.724** ± **0.322**
	EMLP	0.333 ± 0.346	0.987 ± 0.0204	0.992 ± 0.0174	1 ± 0	1 ± 0
	MARL	0.581 ± 0.469	0.734 ± 0.363	0.754 ± 0.34	0.791 ± 0.286	0.871 ± 0.178
	Ours	**1.047** ± **0.159**	**0.404** ± **0.297**	**0.481** ± **0.3**	**0.641** ± **0.232**	**0.719** ± **0.273**
Catch underarm	PPO	1 ± 0.0554	0.239 ± 0.0884	0.383 ± 0.142	0.602 ± 0.231	0.856 ± 0.138
	EMLP	0.702 ± 0.32	0.694 ± 0.32	0.79 ± 0.288	0.862 ± 0.19	0.977 ± 0.0516
	MARL	**1.088** ± **0.0445**	**0.201** ± **0.033**	**0.272** ± **0.0483**	**0.384** ± **0.063**	**0.564** ± **0.0836**
	Ours	**1.098** ± **0.0367**	**0.181** ± **0.0175**	**0.251** ± **0.0177**	**0.336** ± **0.0511**	**0.515** ± **0.109**
Catch abreast	PPO	**1** ± **0.0401**	**0.432** ± **0.203**	0.578 ± 0.183	0.706 ± 0.142	0.914 ± 0.143
	EMLP	0.215 ± 0.321	0.963 ± 0.0831	0.996 ± 0.0088	1 ± 0	1 ± 0
	MARL	0.935 ± 0.495	0.452 ± 0.314	**0.521** ± **0.288**	**0.601** ± **0.257**	**0.7** ± **0.207**
	Ours	**1.236** ± **0.0979**	**0.316** ± **0.0836**	**0.429** ± **0.109**	**0.549** ± **0.0886**	**0.658** ± **0.113**

#### 4.2.2 Comparison of steps-to-threshold

**FIGURE 9:**
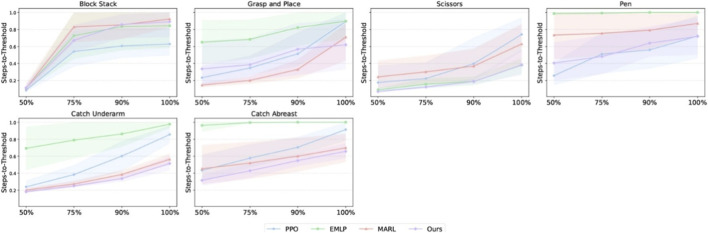
Steps-to-Threshold for asymmetric tasks across five runs with different random seeds. Lower values indicate faster reward acquisition. The error bars indicate 95% confidence intervals.

Figure 9 and the right four columns of Table 4 depict the steps-to-threshold for the asymmetric tasks; Figure 9 shows 95% confidence intervals, whereas Table 4 reports the results as mean ± standard deviation. Although it is not as significant compared to the symmetric tasks, our method shows relatively fast reward acquisition in comparison to the other methods.

### 4.3 Sim-to-real transfer

**FIGURE 10:**
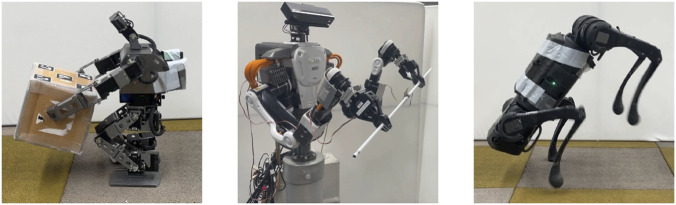
Sim-to-Real Transfer. Left: *Pickup Box* by full-body humanoid robot (Robotis OP3), Center: *Grasp Rod* by upper-body humanoid robot (HIRO), and Right: *Handstand* by quadruped robot (Unitree A1).

As shown in Figure 10, we deployed the trained policies for *Pickup Box* on a full-body humanoid robot, *Rod Grasp* on an upper-body humanoid robot, and *Handstand* on a quadruped robot. For *Pickup Box* and *Rod Grasp*, AR markers were used to estimate the pose of the target object. Videos of the real-world deployment are provided in the Supplementary Material.

To transfer the trained policies to the real robots, we applied domain randomization by adding noise to the observations, actions, and environment dynamics during training. Tables 5–7 summarize the domain randomization parameters used for each robot.

**TABLE 5:** Domain randomization parameters for OP3.

Term	Range
Joint stiffness	[11, 21.5]
Joint damping	[0.2, 0.5]
Body friction	[0.5, 1.0]
Body restitution	[0.0, 0.3]
System delay	[25 ms, 100 ms]
Joint angle offset	( −2.9° , 2.9° )
IMU rotational offset	( 0° , 2° )
Additional weight	[0 kg, 0.5 kg]
Additional weight pos	[-7.5 cm, 7.5 cm]
Random push force	[2 N, 5 N]
Random push force interval	[3 s, 5 s]

**TABLE 6:** Domain randomization parameters for HIRO.

Term	Range
Target positional noise	[-0.025 m, 0.025 m]
Target rotational noise	( −5° , 5° )

**TABLE 7:** Domain randomization parameters for A1.

Term	Range
Joint stiffness	[24, 36]
Joint damping	[0.6, 0.9]
Body friction	[0.4, 2.0]
Body restitution	[0.0, 0.4]
System delay	[10 ms, 40 ms]
Additional link masses	
-Base	[-1 kg, 1 kg]
-Hip	[0, 0.2 kg]
-Thigh	[-0.1 kg, 0.1 kg]
-Calf	[-0.03 kg, 0.03 kg]
-Foot	[-0.01 kg, 0.01 kg]
Link COM offset	[-0.02 m, 0.02 m]
Random push force	[0, 30 N]
Random push force interval	[5 s, 10 s]

We evaluated the real-world success rates of the three tasks. For *Pickup Box*, a trial was considered successful if the robot lifted the box off the ground and held it steadily. In this task, we evaluated two conditions in which the robot was initially positioned 0.5 m and 1.0 m away from the target box. For *Rod Grasp*, success was defined as grasping the rod with both hands and lifting it. For *Handstand*, success was defined as maintaining a foreleg stand for at least 5 seconds. Table 8 presents the real-world success rates over 10 trials for the three tasks. These results suggest that the proposed method learns policies that transfer robustly to real-world settings.

**TABLE 8:** Number of successes over 10 trials for sim-to-real transfer.

Task	PPO	Aug	EMLP	MARL	Ours
Full-body humanoid *Pickup Box*-0.5 m	4/10	7/10	3/10	6/10	8/10
Full-body humanoid *Pickup Box*-1.0 m	0/10	5/10	1/10	7/10	7/10
Upper-body humanoid *Rod Grasp*	7/10	0/10	8/10	9/10	9/10
Quadruped robot *Handstand*	10/10	9/10	6/10	3/10	10/10

## 5 Discussion

### 5.1 Performance on symmetric tasks

We observe that Ours, which combines a multi-agent policy network with a modified symmetric objective, outperforms the other methods on most tasks in terms of both reward acquisition and learning speed. Although the other symmetry-based methods (Aug, EMLP, and MARL) sometimes achieve higher or faster reward acquisition than PPO, they also suffer from substantially degraded performance on specific tasks.

In supervised learning, exploiting morphological symmetry–through data augmentation or symmetric architectural constraints–often leads to consistent performance gains. In DRL, however, such approaches are not necessarily effective to the same extent. A key difference is the highly stochastic nature of DRL: the training dataset (i.e., the experience collected during exploration) is not guaranteed to be symmetrically distributed, even if the underlying environment is perfectly symmetric. Symmetric constraints on the policy network can encourage more balanced behavior, but they cannot fully compensate for asymmetries introduced by stochastic initializations and transitions. For example, in locomotion tasks, the commanded walking direction is typically sampled randomly, and in manipulation tasks, the initial positions of target objects are randomized. These sources of randomness inevitably induce skew in the collected experience, which can conflict with rigid symmetry constraints and, in turn, degrade the performance of symmetry-based methods.

Our method imposes symmetry, but in a different way: it enforces structure at the level of policy construction and uses a joint symmetric objective derived from a MARL-style formulation, where imbalance in the experiences of different “agents” (left and right sides) is expected rather than ignored. This design allows the policy to exploit symmetry when present, while remaining comparatively robust to skewed data.

In addition, the comparison between Ours and MARL highlights the effect of the coupled objective. Across the benchmark tasks, Ours improves both the highest reward and the steps-to-threshold in most cases. As shown in Table 3, Ours achieves a higher reward in 9 out of 10 symmetric tasks, except for *Locomotion* by the full-body humanoid, and a smaller steps-to-threshold at 100% in 9 out of 10 symmetric tasks, except for *Two Catch Underarm* by Bi-DexHands. This trend is further supported by the bootstrap 95% confidence intervals shown in Figures 5, 6. In particular, Ours shows non-overlapping confidence intervals for the highest reward in *CG Humanoid Locomotion*, *CG Humanoid Backflip*, *Pickup Box* by the full-body humanoid, and *Locomotion* by the quadruped robot, suggesting that the improvement is unlikely to be explained solely by seed-level variation. Similarly, for the steps-to-threshold at 100%, non-overlapping confidence intervals are observed in *Pickup Box* by the full-body humanoid, as well as in *Locomotion* and *Handstand* by the quadruped robot.

While splitting the action space into two half-body policies reduces the complexity of the control problem, the MARL objective remains defined at the level of the individual sides. Accordingly, the clipping operation is applied independently to the left and right importance-sampling ratios. In standard PPO, clipping constrains the policy update by restricting the ratio to [1−ϵ, 1+ϵ] . In contrast, when the two sides are clipped independently, the effective ratio of the full policy may deviate more strongly; for example, if both per-side ratios change in the same direction, the combined ratio can reach ${\left(1-\epsilon \right)}^{2}$ or ${\left(1+\epsilon \right)}^{2}$ . This suggests that the decoupled objective may permit more aggressive updates than intended for the full policy.

By clipping the coupled ratio of the full symmetric policy, Ours ties the updates of the two sides together and better matches the structure of the policy itself. We hypothesize that this is one of the main reasons why Ours consistently outperforms MARL.

### 5.2 Robustness to asymmetric environments

Ours remains competitive with PPO even on asymmetric tasks, despite being designed under the assumption of a symmetric MDP. In contrast, EMLP is worse than PPO on five out of the six asymmetric tasks, and the MARL variant typically lies between EMLP and Ours. This behavior is particularly noteworthy given the MARL-style design of Ours. As defined in Equation 9, our observation mapping enforces symmetry only on the intrinsic state, while no permutation-based symmetry constraint is imposed on the extrinsic state.

Although the advantage of the proposed method on asymmetric tasks is smaller than that observed on symmetric tasks, Ours does not degrade substantially and maintains performance at a level comparable to or better than the baseline PPO, whereas EMLP and MARL suffer large performance drops on some tasks. While Ours underperforms PPO on *Block Stack*, the gap is generally modest and is often accompanied by overlapping confidence intervals, making the difference inconclusive given the current number of random seeds. These results suggest that the coupled objective primarily improves robustness against asymmetric environments rather than merely exploiting symmetry-specific structure. In practical settings, it is rare for the environment and task to strictly satisfy the symmetric MDP assumptions. Our findings indicate that the proposed method can perform well across a wide range of tasks, as long as the robot itself exhibits morphological symmetry, even when the surrounding environment is asymmetric.

## 6 Conclusion

In this work, we proposed a symmetry-assisted DRL framework for morphologically symmetric robots that exploits left–right reflection symmetry at both the policy and training objective levels. We modeled the environment as a symmetric MDP equipped with symmetry operators on the state, action, and configuration spaces, and constructed a full-body policy from a single base policy acting on one side of the robot. By mirroring observations and actions through these operators, our method guarantees an equivariant policy structure while reducing the effective action dimension and retaining the simplicity of standard MLP architectures.

To align the optimization procedure with this structural symmetry, we further introduced a symmetric PPO objective based on a coupled importance-sampling ratio for the full policy. Unlike MAPPO-style MARL formulations, which share parameters but treat the left and right sides as separate agents with per-side clipping, our objective updates both sides in a coordinated fashion that is consistent with the underlying symmetry. We compared this framework with four baselines–PPO, data augmentation, EMLP, and a MARL variant–on ten symmetric and six asymmetric tasks across five robotic platforms, including simulated and real humanoid robots, a quadruped, and dexterous multi-fingered hands.

There are several directions for future work. First, extending the framework to more general symmetry groups beyond simple reflections (e.g., cyclic or permutation groups for multi-legged or modular robots) could further broaden its applicability. Second, combining our symmetric PPO objective with richer value-function architectures or model-based components may improve sample efficiency in more complex tasks. Second, while our focus in this work is on symmetry-assisted DRL, combining the proposed objective with richer value-function architectures or model-based components may further improve sample efficiency in more complex tasks. In particular, for contact-rich robotic systems where accurate analytical contact-dynamics models are available, model-based trajectory optimization methods such as DDP (Singh et al., 2023) offer a complementary route to improving sample efficiency. Likewise, for long-horizon or multi-stage robotic tasks, hierarchical model-based structures such as primitive-based planning (Sun et al., 2025) may provide useful inductive bias for improving data efficiency and execution reliability. Finally, a deeper theoretical analysis of the interplay among symmetry, data skew, and optimization dynamics could lead to principled criteria for choosing among architectural, objective-level, and hybrid symmetry-exploiting methods in practical robotic systems.

## Data Availability

The raw data supporting the conclusions of this article will be made available by the authors, without undue reservation.

## Footnotes

1
https://github.com/isaac-sim/IsaacGymEnvs

2
https://emanual.robotis.com/docs/en/software/dynamixel/dynamixel_sdk/overview/
