# Correction: Morphological symmetry-aware generalized policy network for deep reinforcement learning

**DOI:** 10.3389/frobt.2026.1904541

**Published:** 2026-06-29

**Authors:** Frontiers Production Office

**Affiliations:** Frontiers Media SA, Lausanne, Switzerland

**Keywords:** deep reinforcement learning, humanoid robots, legged locomotion, manipulation, morphological symmetry, quadruped robots

There was a mistake in Figures 6–10. The images for the Figures 6–10 were interchanged incorrectly, causing a mismatch between the images and the captions. Additionally, the original Figure 10 is now renamed as Figure 6 and the following figures have been renumbered accordingly, and their in-text citation has been corrected.

**FIGURE 6:**
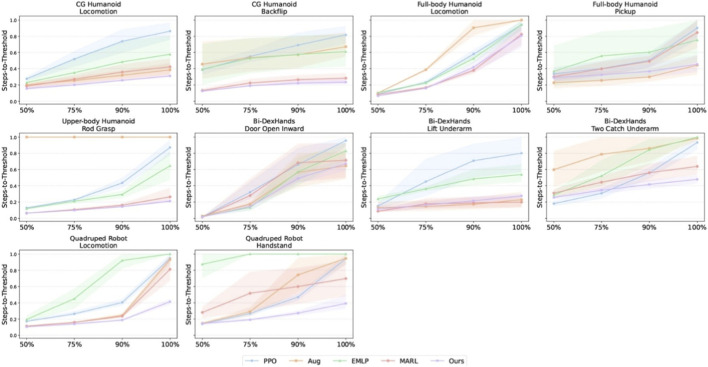
Steps-to-Threshold for symmetric tasks across five runs with different random seeds. Lower values indicate faster reward acquisition. The error bars indicate 95% confidence intervals.

**FIGURE 7:**
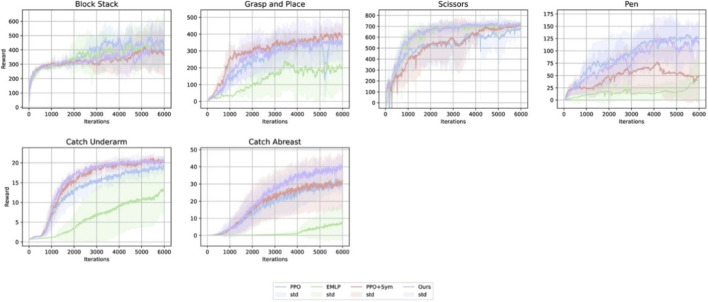
Reward curves for asymmetric tasks across five runs with different random seeds. The light-colored areas indicate 95% confidence intervals.

**FIGURE 8:**
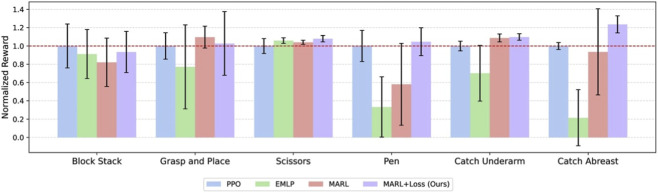
Highest reward comparison for asymmetric tasks across five runs with different random seeds. The values are normalized by the value of PPO. The error bars indicate 95% confidence intervals.

**FIGURE 9:**
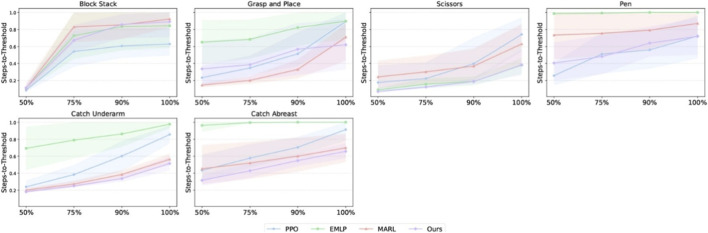
Steps-to-Threshold for asymmetric tasks across five runs with different random seeds. Lower values indicate faster reward acquisition. The error bars indicate 95% confidence intervals.

**FIGURE 10:**
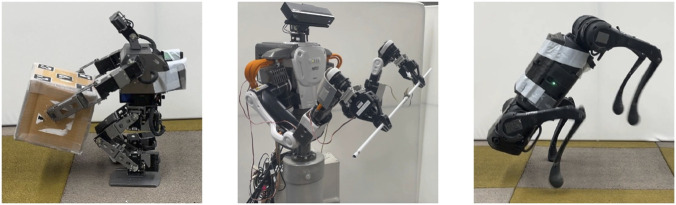
Sim-to-Real Transfer. Left: *Pickup Box* by full-body humanoid robot (Robotis OP3), Center: *Grasp Rod* by upper-body humanoid robot (HIRO), and Right: *Handstand* by quadruped robot (Unitree A1).

Finally, in Section 4.1.2 *Steps-to-threshold comparison,* the sentence “Table 3 presents the normalized number of iterations required to reach given fractions of the final PPO return on symmetric tasks” is now corrected to “Figure 6 and the right four columns of Table 3 depict the steps-to-threshold for the symmetric tasks; Figure 6 shows 95% confidence intervals, whereas Table 3 reports the results as mean ± standard deviation”.

The original article has been updated.

