# Supplementary material for: Morphological symmetry-aware generalized policy network for deep reinforcement learning
Source: Front Robot AI. 2026 May 13;13:1816301. doi: 10.3389/frobt.2026.1816301 (PMC13213176; doi:10.3389/frobt.2026.1816301)
Supplement: Supplementary file 1 [file Supplementaryfile1.pdf]

frontiers

Supplementary Material

1 IMPLEMENTATION DETAILS

In this material, we detail the implementation details for the tasks in the experiment, such as state space, action space, their corresponding symmetry operators, and hyperparameters of DRL.

1.1 Table Conventions

Before presenting the robot and task details, we first explain how to interpret the tables. We provide the joint list and intrinsic state variables for each robot, along with the extrinsic state variables for each task.

Each joint list includes the joint ID, joint name, side label, the joint-wise flipping operator F_{joint}, and the joint-wise permutation operator P_{joint}. The side label indicates whether the joint belongs to the left or right joint-configuration space, $\mathcal{Q}_l$ or $\mathcal{Q}_r$. For F_{joint}, a value of -1 indicates sign inversion of the corresponding joint state, while a value of 1 indicates that the state remains unchanged. P_{joint} specifies the target joint ID under the permutation.

Each intrinsic and extrinsic state table includes the environment-related state vectors, the flipping operator $F_{\text{intr/extr}}$, and the permutation operator $P_{\text{intr/extr}}$. In $F_{\text{intr/extr}}$, $\text{flip}(\cdot)$ denotes the sign inversion of the state-vector elements at the specified indices. In $P_{\text{intr/extr}}$, $\text{to}(\cdot)$ denotes the swapping of the corresponding vectors.

The action symmetry operators $\mathcal{M}_a$ are defined based on the joint lists, whereas the state symmetry operators $\mathcal{M}_s$ are defined based on the intrinsic and extrinsic state tables. For MARL-based methods, the split operator $\mathcal{M}_{\text{split}}$ is determined by the side labels in the joint lists.

1.2 CG Humanoid

1.2.1 Intrinsic state space

CG humanoid has 21 joints as shown in Table S1. The intrinsic state of the humanoid is shown in Table S2

1.2.2 Action Space and Actuation

Let $\mathbf{a} \in \mathbb{R}^{21}$ denote the action, where each element corresponds to one actuated DoF of the humanoid. The action is interpreted as a normalized actuation command and converted into a joint force command through

$$\boldsymbol{\tau} = \tau^{\max}\mathbf{a}, \tag{S1}$$

where $\tau^{\max}$ is the actuator effort limit obtained from the humanoid asset. The robot is actuated by the calculated joint force at 60 Hz.

1.2.3 Task Details

Locomotion**:** Table S3 shows the extrinsic states and their corresponding symmetry operators used for state symmetrization in the *Locomotion* task with CG Humanoid. The target direction is fixed as the forward vector (the $+x$ direction), and the agent learns to run toward it.

Table S1. Joint list and symmetric operators of CG Humanoid.

ID	Name	Side	F_{joint}	P_{joint}
0	Abdomen Z	L+R	-1	0
1	Abdomen Y	L+R	1	1
2	Abdomen X	L+R	-1	2
3	Right Hip X	R	1	9
4	Right Hip Z	R	1	10
5	Right Hip Y	R	1	11
6	Right Knee	R	1	12
7	Right Ankle Y	R	1	13
8	Right Ankle X	R	-1	14
9	Left Hip X	L	1	3
10	Left Hip Z	L	1	4
11	Left Hip Y	L	1	5
12	Left Knee	L	1	6
13	Left Ankle Y	L	1	7
14	Left Ankle X	L	-1	8
15	Right Shoulder1	R	1	18
16	Right Shoulder2	R	1	19
17	Right Elbow	R	1	20
18	Left Shoulder1	L	1	15
19	Left Shoulder2	L	1	16
20	Left Elbow	L	1	17
Total	21			

Table S2. Intrinsic states and symmetry operators of CG Humanoid

Symbol	Dimension	Description	F_{intr}	P_{intr}
h_t	1	Torso height	1	-
$\mathbf{v}_{\text{loc}}$	3	Torso linear velocity	$\text{flip}(1)$	-
$\boldsymbol{\omega}_{\text{loc}}$	3	Torso angular velocity	$\text{flip}(0, 1)$	-
$\mathbf{R}_{\text{root}}$	4	Torso rotation quaternion	$\text{flip}(1, 3)$	-
$\mathbf{q}$	21	Joint positions	F_{joint}	P_{joint}
$\dot{\mathbf{q}}$	21	Joint velocities	F_{joint}	P_{joint}
$\boldsymbol{\tau}$	21	Joint torque	F_{joint}	P_{joint}
$\mathbf{f}_{\text{L}}$	6	Foot force/torque sensor	$\text{flip}(1, 3, 5)$	$\text{to}(\mathbf{f}_{\text{R}})$
$\mathbf{f}_{\text{R}}$	6	Foot force/torque sensor	$\text{flip}(1, 3, 5)$	$\text{to}(\mathbf{f}_{\text{L}})$
$\mathbf{a}_{\text{prev}}$	21	Previous action	F_{joint}	P_{joint}
Total	106			

Table S3. Extrinsic states and symmetry operators of *Locomotion* by CG Humanoid

Symbol	Dimension	Description	F_{extr}	P_{extr}
θ	1	Relative angle to the target direction	$\text{flip}(0)$	-
u	1	Torso upper vector projection	-	-
d	1	Torso forward vector projection	-	-
Total	3			

***Backflip*:** Table S4 shows the extrinsic states and their corresponding symmetry operators used for state symmetrization in the *Backflip* task with CG Humanoid. The backflip motion is divided into five phases: standing, rotated by $90°$, rotated by $180°$, rotated by $270°$, and landing. The extrinsic state is represented by a five-dimensional one-hot vector $\mathbf{o}$ that indicates the current phase of the backflip.

Table S4. Extrinsic states and symmetry operators of *Backflip* by CG Humanoid

Symbol	Dimension	Description	F_{extr}	P_{extr}
$\mathbf{o}$	5	One-hot vector for backflip phase	-	-
Total	5			

1.2.4 Training Parameters

Table S5 summarizes the training hyperparameters used for the CG Humanoid tasks.

Table S5. Hyperparameters in training of CG Humanoid

Parameter	Value
Actor hidden layers	[400, 200, 100]
Critic hidden layer	[400, 200, 100]
Activation	ELU
Clip range	0.2
Discount factor	0.99
GAE discount factor	0.95
Desired KL divergence	0.008
Learning rate	5×10^{-4}
Number of Environments	4096
Steps per iteration	32
Training iterations	
-*Locomotion*	1000
-*Backflip*	3000

1.3 Full-body Humanoid

1.3.1 Intrinsic state space

The full-body humanoid robot (OP3, ROBOTIS Corp.) has 20 joints, as shown in Table S6. The intrinsic state is shown in Table S7. The intrinsic states from the previous five time steps are stacked and fed into the policy.

1.3.2 Action Space and Actuation

Let $\mathbf{a} \in \mathbb{R}^{20}$ denote the action vector, whose elements correspond to the actuated DoFs of the robot. The action is interpreted as a normalized target joint position. The commanded target joint position is then computed as

$$\tilde{\mathbf{q}} = 0.8\tilde{\mathbf{q}}_{\mathrm{prev}} + 0.2\mathrm{unnorm}(\mathbf{a}), \tag{S2}$$

where $\tilde{\mathbf{q}}$ is the current target joint position, $\tilde{\mathbf{q}}_{\mathrm{prev}}$ is the target joint position at the previous time step, and $\mathrm{unnorm}(\cdot)$ maps the normalized action to the joint limits. Each joint is controlled by a PD controller at 40 Hz.

1.3.3 Task Details

***Locomotion*:** Table S8 presents the extrinsic states and their corresponding symmetry operators used for state symmetrization in the *Locomotion* task with full-body humanoid robot. The target heading direction is randomly sampled during training and provided by a higher-level controller during inference, and the agent is trained to run toward it.

Table S6. Joint list of Full-body Humanoid

ID	Name	Side	F_{joint}	P_{joint}
0	Head Pan	L+R	-1	0
1	Head Tilt	L+R	1	1
2	Left Hip Yaw	L	-1	11
3	Left Hip Roll	L	-1	12
4	Left Hip Pitch	L	-1	13
5	Left Knee	L	-1	14
6	Left Ankle Pitch	L	-1	15
7	Left Ankle Roll	L	-1	16
8	Left Shoulder Pitch	L	-1	17
9	Left Shoulder Roll	L	-1	18
10	Left Elbow	L	-1	19
11	Right Hip Yaw	R	-1	2
12	Right Hip Roll	R	-1	3
13	Right Hip Pitch	R	-1	4
14	Right Knee	R	-1	5
15	Right Ankle Pitch	R	-1	6
16	Right Ankle Roll	R	-1	7
17	Right Shoulder Pitch	R	-1	8
18	Right Shoulder Roll	R	-1	9
19	Right Elbow	R	-1	10
Total	20			

Table S7. Intrinsic states and symmetry operators of Full-body Humanoid

Symbol	Dimension	Description	F_{intr}	P_{intr}
$\boldsymbol{\omega}_{\text{loc}}$	3	Local angular velocity	$\text{flip}(0, 2)$	-
$\mathbf{g}_{\text{loc}}$	3	Projected gravity direction	$\text{flip}(1)$	-
$\mathbf{q}$	18	Joint positions	F_{joint}	P_{joint}
$\tilde{\mathbf{q}}_{\text{prev}}$	18	Previous joint target positions	F_{joint}	P_{joint}
Total	42			

Table S8. Extrinsic states and symmetry operators of *Locomotion* by Full-body Humanoid

Symbol	Dimension	Description	F_{extr}	P_{extr}
$\mathbf{d}_{\text{loc}}$	3	Projected target heading direction	$\text{flip}(1)$	-
Total	3			

Table S9. Extrinsic states and symmetry operators of *Pickup Box* by Full-body Humanoid

Symbol	Dimension	Description	F_{extr}	P_{extr}
$\mathbf{p}_{\text{box}}$	3	Position of the box	$\text{flip}(1)$	-
$\mathbf{u}_{\text{box}}$	3	Up vector (+z) of the box	$\text{flip}(1)$	-
$\mathbf{f}_{\text{box}}$	3	Forward vector (+x) of the box	$\text{flip}(1)$	-
Total	9			

The extrinsic states from the previous five time steps are stacked and fed into the policy.

***Pickup Box*:** Table S9 presents the extrinsic states and their corresponding symmetry operators used for state symmetrization in the *Pickup Box* task with the full-body humanoid robot. The extrinsic state is defined by the position of the target box and its orientation, where the orientation is represented by the up and forward vectors. The extrinsic states from the previous five time steps are stacked and provided as input to the policy.

1.3.4 Training Parameters

Table S10 summarizes the training hyperparameters used for the Full-body Humanoid tasks.

Table S10. Hyperparameters in training of Full-body Humanoid

Parameter	Value
Actor hidden layers	[256, 128, 64]
Critic hidden layer	[256, 128, 64]
Activation	ELU
Clip range	0.2
Discount factor	0.99
GAE discount factor	0.95
Desired KL divergence	0.008
Learning rate	5×10^{-4}
Number of Environments	4096
Steps per iteration	32
Training iterations	
-*Locomotion*	1500
-*Pickup Box*	5000

1.4 Upper-body Humanoid

1.4.1 Intrinsic State Space

Upper-body Humanoid Robot (HIRO, Kawada Robotics Corp. and RH-P12-RN, Robotis Corp.) has 15 joints, as shown in Table S11. The intrinsic state is shown in Table S12.

1.4.2 Action Space and Actuation

Let $\mathbf{a} \in \mathbb{R}^{15}$ denote the action vector, whose elements correspond to the actuated DoFs of the robot. The action is interpreted as the deviation of joint position from the previous time step. The commanded target joint position is then computed as

$$\tilde{\mathbf{q}} = \mathbf{q}_{\text{prev}} + \alpha \mathbf{a}, \tag{S3}$$

where $\tilde{\mathrm{q}}$ is the current target joint position, $\mathbf{q}_{\text{prev}}$ is the joint position at the previous time step, and α is coefficient of joint deviation. Each joint is controlled by a PD controller at 10 Hz.

1.4.3 Task Details

Grasp Rod: Table S13 presents the extrinsic states and their corresponding symmetry operators used for state symmetrization in the *Grasp Rod* task with the upper-body humanoid robot. The extrinsic state is defined by the center position and axis direction of the rod.

1.4.4 Training Parameters

Table S14 summarizes the training hyperparameters used for the Upper-body Humanoid tasks.

1.5 Bi-DexHands

1.5.1 Intrinsic state space

Bi-DexHands has 40 joints and 12 DoFs of base poses as shown in Table S15. For convenience, we define the symmetric operators both for joints (F_{joint} and P_{joint}) and base poses (F_{base} and P_{base}) and represent

Table S11. Joint list of Upper-body Humanoid

ID	Name	Side	F_{joint}	P_{joint}
0	Chest Joint	L+R	-1	0
1	Left Arm Joint0	L	-1	8
2	Left Arm Joint1	L	1	9
3	Left Arm Joint2	L	1	10
4	Left Arm Joint3	L	-1	11
5	Left Arm Joint4	L	1	12
6	Left Arm Joint5	L	-1	13
7	Left Hand	L	1	14
8	Rarm Joint0	R	-1	1
9	Rarm Joint1	R	1	2
10	Rarm Joint2	R	1	3
11	Rarm Joint3	R	-1	4
12	Rarm Joint4	R	1	5
13	Rarm Joint5	R	-1	6
14	Right Hand	R	1	7
Total	15			

Table S12. Intrinsic states and symmetry operators of Upper-body Humanoid

Symbol	Dimension	Description	F_{intr}	P_{intr}
$\mathbf{q}$	15	Joint positions	F_{joint}	P_{joint}
Total	15			

Table S13. Extrinsic states and symmetry operators of *Grasp Rod* by Upper-body Humanoid

Symbol	Dimension	Description	F_{extr}	P_{extr}
$\mathbf{p}_{\text{rod}}$	3	Position of the rod center	$\text{flip}(1)$	-
$\mathbf{a}_{\text{rod}}$	3	Axis direction of the rod	$\text{flip}(1)$	-
Total	6			

Table S14. Hyperparameters in training of Upper-body Humanoid

Parameter	Value
Actor hidden layers	[512, 256, 64]
Critic hidden layer	[512, 256, 64]
Activation	ELU
Clip range	0.2
Discount factor	0.99
GAE discount factor	0.95
Desired KL divergence	0.008
Learning rate	5×10^{-4}
Number of Environments	4096
Steps per iteration	8
Training iterations	3000

the symmetric operator for entire DoFs as $F_{\text{joint}} \circ F_{\text{base}}$ and $P_{\text{joint}} \circ P_{\text{base}}$. The intrinsic state is shown in Table S16.

1.5.2 Action Space and Actuation

Let $\mathbf{a} \in \mathbb{R}^{52}$ denote the action vector, whose elements correspond to the actuated joints and the base wrenches of the robot. The subvector $\mathbf{a}_{0:39}$ is interpreted as a normalized target joint position, and the

commanded target joint position is given by

$$\tilde{\mathbf{q}} = \mathrm{unnorm}(\mathbf{a}_{0:39}), \tag{S4}$$

where $\tilde{\mathbf{q}}$ denotes the current target joint position, and $\mathrm{unnorm}(\cdot)$ maps the normalized action to the joint limits. Each joint is controlled by a PD controller at 60 Hz.

The subvector $\mathbf{a}_{40:52}$ represents the forces and torques applied to the base links of the left and right hands:

$$\boldsymbol{f}_{\mathrm{L}} = f_{\max}\mathbf{a}_{40:42}, \tag{S5}$$
$$\boldsymbol{\tau}_{\mathrm{L}} = \tau_{\max}\mathbf{a}_{43:45}, \tag{S6}$$
$$\boldsymbol{f}_{\mathrm{R}} = f_{\max}\mathbf{a}_{46:48}, \tag{S7}$$
$$\boldsymbol{\tau}_{\mathrm{R}} = \tau_{\max}\mathbf{a}_{49:52}. \tag{S8}$$

Here, $\boldsymbol{f}_{\mathrm{L}}, \boldsymbol{f}_{\mathrm{R}} \in \mathbb{R}^3$ are the forces applied to the left and right hand bases, respectively, $\boldsymbol{\tau}_{\mathrm{L}}, \boldsymbol{\tau}_{\mathrm{R}} \in \mathbb{R}^3$ are the corresponding torques, and $f_{\max}, \tau_{\max} \in \mathbb{R}$ are the force and torque limits.

1.5.3 Task Details

***Door Open Inward*:** Table S17 presents the extrinsic states and their corresponding symmetry operators used for state symmetrization in the *Door Open Inward* task with Bi-DexHands. The extrinsic state contains the position of the handle of doors.

***Lift Underarm*:** Table S18 presents the extrinsic states and their corresponding symmetry operators used for state symmetrization in the *Lift Underarm* task with Bi-DexHands. The extrinsic state is defined by the position, orientation, linear velocity, and angular velocity of the pot, together with the positions of the pot handles.

***Two Catch Underarm*:**

Table S19 presents the extrinsic states and their corresponding symmetry operators used for state symmetrization in the *Two Catch Underarm* task with Bi-DexHands. The extrinsic state consists of the positions, orientations, linear velocities, and angular velocities of the two balls, as well as their goal positions and orientations.

1.5.4 Training Parameters

Table S20 summarizes the training hyperparameters used for the Bi-DexHands tasks.

1.6 Quadruped Robot

1.6.1 Intrinsic state space

Quadruped Robot (A1, Unitree Corp.) has 12 joints, as shown in Table S21. The intrinsic state is shown in Table S22.

1.6.2 Action Space and Actuation

Let $\mathbf{a} \in \mathbb{R}^{12}$ denote the action vector, whose elements correspond to the actuated DoFs of the robot. The action is interpreted as a target joint position. The commanded target joint position is then computed as

$$\tilde{\mathbf{q}} = \mathbf{q}_{\mathrm{def}} + \alpha\mathbf{a}, \tag{S9}$$

where $\tilde{\mathbf{q}}$ is the current target joint position, $\mathbf{q}_{\text{def}}$ is the default joint position, and α is action coefficient. Each joint is controlled by a PD controller at 50 Hz.

1.6.3 Task Details

***Locomotion*:** Table S23 presents the extrinsic states and their corresponding symmetry operators used for state symmetrization in the *Locomotion* task with the quadruped robot. The target linear and angular velocities are randomly sampled during training and provided by a higher-level controller during inference, and the agent is trained to move according to these commands.

***Handstand*:** No extrinsic state is used for the *Handstand* task with the quadruped robot. The agent is trained to maintain a stable handstand while minimizing body sway.

1.6.4 Training Parameters

Table S24 summarizes the training hyperparameters used for the Quadruped robot tasks.

1.7 Asymmetric Tasks

For asymmetric tasks, we use Bi-DexHands environments. The intrinsic states and action space are detailed in Section 1.5.1 and 1.5.2.

1.7.1 *Block Stack* Details

Table S25 presents the extrinsic states and their corresponding symmetry operators used for state symmetrization in the *Block Stack* task.

1.7.2 *Grasp and Place* Details

Table S26 presents the extrinsic states and their corresponding symmetry operators used for state symmetrization in the *Grasp and Place* task.

1.7.3 *Scissors* Details

Table S27 presents the extrinsic states and their corresponding symmetry operators used for state symmetrization in the *Scissors* task.

1.7.4 *Pen* Details

Table S28 presents the extrinsic states and their corresponding symmetry operators used for state symmetrization in the *Pen* task.

1.7.5 *Catch Underarm* and *Catch Abreast* Details

Table S29 presents the extrinsic states and their corresponding symmetry operators used for state symmetrization in the *Catch Underarm* and *Catch Abreast* task.

1.7.6 Training Parameters

Table S30 summarizes the training hyperparameters used for the asymmetric tasks.

Table S15. Joint list of Bi-DexHands

ID	Name	Side	F_{joint}	P_{joint}
0	Left Wrist Joint0	L	1	20
1	Left Wrist Joint1	L	1	21
2	Left First Finger Joint0	L	1	22
3	Left First Finger Joint1	L	1	23
4	Left First Finger Joint2	L	1	24
5	Left Middle Finger Joint0	L	1	25
6	Left Middle Finger Joint1	L	1	26
7	Left Middle Finger Joint2	L	1	27
8	Left Ring Finger Joint0	L	1	28
9	Left Ring Finger Joint1	L	1	29
10	Left Ring Finger Joint2	L	1	30
11	Left Little Finger Joint0	L	1	31
12	Left Little Finger Joint1	L	1	32
13	Left Little Finger Joint2	L	1	33
14	Left Little Finger Joint3	L	1	34
15	Left Thumb Joint0	L	1	35
16	Left Thumb Joint1	L	1	36
17	Left Thumb Joint2	L	1	37
18	Left Thumb Joint3	L	1	38
19	Left Thumb Joint4	L	1	39
20	Right Wrist Joint0	R	1	0
21	Right Wrist Joint1	R	1	1
22	Right First Finger Joint0	R	1	2
23	Right First Finger Joint1	R	1	3
24	Right First Finger Joint2	R	1	4
25	Right Middle Finger Joint0	R	1	5
26	Right Middle Finger Joint1	R	1	6
27	Right Middle Finger Joint2	R	1	7
28	Right Ring Finger Joint0	R	1	8
29	Right Ring Finger Joint1	R	1	9
30	Right Ring Finger Joint2	R	1	10
31	Right Little Finger Joint0	R	1	11
32	Right Little Finger Joint1	R	1	12
33	Right Little Finger Joint2	R	1	13
34	Right Little Finger Joint3	R	1	14
35	Right Thumb Joint0	R	1	15
36	Right Thumb Joint1	R	1	16
37	Right Thumb Joint2	R	1	17
38	Right Thumb Joint3	R	1	18
39	Right Thumb Joint4	R	1	19
ID	Name	Side	F_{base}	P_{base}
40	Left Base Force X	L	1	46
41	Left Base Force Y	L	-1	47
42	Left Base Force Z	L	1	48
43	Left Base Torque X	L	-1	49
44	Left Base Torque Y	L	1	50
45	Left Base Torque Z	L	-1	51
46	Right Base Force X	R	1	40
47	Right Base Force Y	R	-1	41
48	Right Base Force Z	R	1	42
49	Right Base Torque X	R	-1	43
50	Right Base Torque Y	R	1	44
51	Right Base Torque Z	R	-1	45
Total	52			

Table S16. Intrinsic states and symmetry operators of Bi-DexHands

Symbol	Dimension	Description	F_{intr}	P_{intr}
$\mathbf{q}$	48	Joint positions	F_{joint}	P_{joint}
$\dot{\mathbf{q}}$	48	Joint velocities	F_{joint}	P_{joint}
$\boldsymbol{\tau}$	48	Joint torques	F_{joint}	P_{joint}
$\{\mathbf{f}_k^L\}_{k=0}^4$	65	Left-hand fingertip states	*1	$\text{to}(\{\mathbf{f}_k^R\})$
$\{\mathbf{f}_k^R\}_{k=0}^4$	65	Right-hand fingertip states	*1	$\text{to}(\{\mathbf{f}_k^L\})$
$\{\mathbf{s}_k^L\}_{k=0}^4$	30	Left-hand fingertip force	*2	$\text{to}(\{\mathbf{s}_k^R\})$
$\{\mathbf{s}_k^R\}_{k=0}^4$	30	Right-hand fingertip force	*2	$\text{to}(\{\mathbf{s}_k^L\})$
$\mathbf{p}$	12	Hand base poses	F_{base}	P_{base}
$\mathbf{a}_{\text{prev}}$	26	Previous action	$F_{\text{joint}} \circ F_{\text{base}}$	$P_{\text{joint}} \circ P_{\text{base}}$
Total	42			

*1: The fingertip state $\mathbf{f}_k^{\{L|R\}} \in \mathbb{R}^{13}$ contains the position, orientation (represented as a quaternion), linear velocity, and angular velocity of each fingertip. The flipping operator for a single fingertip is $\text{flip}(1, 4, 6, 8, 10, 12)$.
*2: The fingertip force state $\mathbf{s}_k^{\{L|R\}}$ contains the force and torque at each fingertip. The flipping operator for a single fingertip is $\text{flip}(1, 3, 5)$.

Table S17. Extrinsic states and symmetry operators of *Door Open Inward* by Bi-DexHands

Symbol	Dimension	Description	F_{extr}	P_{extr}
$\mathbf{p}_{\text{LHandle}}$	3	Left door-handle position	$\text{flip}(1)$	$\text{to}(\mathbf{p}_{\text{RHandle}})$
$\mathbf{p}_{\text{RHandle}}$	3	Right door-handle position	$\text{flip}(1)$	$\text{to}(\mathbf{p}_{\text{LHandle}})$
Total	6			

Table S18. Extrinsic states and symmetry operators of *Lift Underarm* by Bi-DexHands

Symbol	Dimension	Description	F_{extr}	P_{extr}
$\mathbf{p}_{\text{pot}}$	3	Pot position	$\text{flip}(1)$	-
$\mathbf{R}_{\text{pot}}$	4	Pot orientation quaternion	$\text{flip}(1, 3)$	-
$\mathbf{v}_{\text{pot}}$	3	Pot linear velocity	$\text{flip}(1)$	-
$\boldsymbol{\omega}_{\text{pot}}$	3	Pot angular velocity	$\text{flip}(0, 2)$	-
$\mathbf{p}_{\text{LHandle}}$	3	Left pot-handle position	$\text{flip}(1)$	$\text{to}(\mathbf{p}_{\text{RHandle}})$
$\mathbf{p}_{\text{RHandle}}$	3	Right pot-handle position	$\text{flip}(1)$	$\text{to}(\mathbf{p}_{\text{LHandle}})$
Total	19			

Table S19. Extrinsic states and symmetry operators of *Two Catch Underarm* by Bi-DexHands

Symbol	Dimension	Description	F_{extr}	P_{extr}
$\mathbf{p}_{\text{LBall}}$	3	Left ball position	$\text{flip}(1)$	$\text{to}(\mathbf{p}_{\text{RBall}})$
$\mathbf{R}_{\text{LBall}}$	4	Left ball orientation quaternion	$\text{flip}(1, 3)$	$\text{to}(\mathbf{R}_{\text{RBall}})$
$\mathbf{v}_{\text{LBall}}$	3	Left ball linear velocity	$\text{flip}(1)$	$\text{to}(\mathbf{v}_{\text{RBall}})$
$\boldsymbol{\omega}_{\text{LBall}}$	3	Left ball angular velocity	$\text{flip}(0, 2)$	$\text{to}(\boldsymbol{\omega}_{\text{RBall}})$
$\mathbf{p}_{\text{LGoal}}$	3	Left goal position	$\text{flip}(1)$	$\text{to}(\mathbf{p}_{\text{RGoal}})$
$\mathbf{R}_{\text{LGoal}}$	4	Left goal orientation quaternion	$\text{flip}(1, 3)$	$\text{to}(\mathbf{R}_{\text{RGoal}})$
$\hat{\mathbf{R}}_{\text{LGoal}}$	4	Left goal orientation quaternion in left ball frame	$\text{flip}(1, 3)$	$\text{to}(\hat{\mathbf{R}}_{\text{RGoal}})$
$\mathbf{p}_{\text{RBall}}$	3	Right ball position	$\text{flip}(1)$	$\text{to}(\mathbf{p}_{\text{LBall}})$
$\mathbf{R}_{\text{RBall}}$	4	Right ball orientation quaternion	$\text{flip}(1, 3)$	$\text{to}(\mathbf{R}_{\text{LBall}})$
$\mathbf{v}_{\text{RBall}}$	3	Right ball linear velocity	$\text{flip}(1)$	$\text{to}(\mathbf{v}_{\text{LBall}})$
$\boldsymbol{\omega}_{\text{RBall}}$	3	Right ball angular velocity	$\text{flip}(0, 2)$	$\text{to}(\boldsymbol{\omega}_{\text{LBall}})$
$\mathbf{p}_{\text{RGoal}}$	3	Right goal position	$\text{flip}(1)$	$\text{to}(\mathbf{p}_{\text{LGoal}})$
$\mathbf{R}_{\text{RGoal}}$	4	Right goal orientation quaternion	$\text{flip}(1, 3)$	$\text{to}(\mathbf{R}_{\text{LGoal}})$
$\hat{\mathbf{R}}_{\text{RGoal}}$	4	Right goal orientation quaternion in right ball frame	$\text{flip}(1, 3)$	$\text{to}(\hat{\mathbf{R}}_{\text{LGoal}})$
Total	26			

Table S20. Hyperparameters in training of Bi-DexHands

Parameter	Value
Actor hidden layers	[512, 256, 128]
Critic hidden layer	[512, 256, 128]
Activation	ELU
Clip range	0.2
Discount factor	0.96
GAE discount factor	0.95
Desired KL divergence	0.016
Learning rate	3×10^{-4}
Number of Environments	2048
Steps per iteration	8
Training iterations	
-*Two Catch Underarm*	12000
- Others	6000

Table S21. Joint list of Quadruped Robot

ID	Name	Side	F_{joint}	P_{joint}
0	FL Hip Joint	L	-1	3
1	FL Thigh Joint	L	1	4
2	FL Calf Joint	L	1	5
3	FR Hip Joint	R	-1	0
4	FR Thigh Joint	R	1	1
5	FR Calf Joint	R	1	2
6	RL Hip Joint	L	-1	9
7	RL Thigh Joint	L	1	10
8	RL Calf Joint	L	1	11
9	RR Hip Joint	R	-1	6
10	RR Thigh Joint	R	1	7
11	RR Calf Joint	R	1	8
Total	12			

Table S22. Intrinsic states and symmetry operators of Quadruped robot

Symbol	Dimension	Description	F_{intr}	P_{intr}
$\mathbf{v}$	3	Base linear velocity	$\text{flip}(1)$	-
$\boldsymbol{\omega}$	3	Base angular velocity	$\text{flip}(0, 2)$	-
$\mathbf{g}$	3	Projected gravity direction	$\text{flip}(1)$	-
$\mathbf{q}$	12	Joint positions	F_{joint}	P_{joint}
$\dot{\mathbf{q}}$	12	Joint velocities	F_{joint}	P_{joint}
$\mathbf{a}_{\text{prev}}$	12	Previous actions	F_{joint}	P_{joint}
Total	45			

Table S23. Extrinsic states and symmetry operators of *Locomotion* by Full-body Humanoid

Symbol	Dimension	Description	F_{extr}	P_{extr}
$\mathbf{v}_{\text{target}}$	2	Target linear velocity	$\text{flip}(1)$	-
$\boldsymbol{\omega}_{\text{target}}$	1	Target angular	$\text{flip}(0)$	-
Total	3			

Table S24. Hyperparameters in training of Quadruped robot

Parameter	Value
Actor hidden layers	[256, 128, 64]
Critic hidden layer	[256, 128, 64]
Activation	ELU
Clip range	0.2
Discount factor	0.99
GAE discount factor	0.95
Desired KL divergence	0.008
Learning rate	3×10^{-4}
Number of Environments	4096
Steps per iteration	24
Training iterations	1000

Table S25. Extrinsic states and symmetry operators of *Block Stack*

Symbol	Dimension	Description	F_{extr}
$\mathbf{p}_{\text{LBlock}}$	3	Left block position	$\text{flip}(1)$
$\mathbf{R}_{\text{LBlock}}$	4	Left block orientation quaternion	$\text{flip}(1,3)$
$\mathbf{v}_{\text{LBlock}}$	3	Left block linear velocity	$\text{flip}(1)$
$\boldsymbol{\omega}_{\text{LBlock}}$	3	Left block angular velocity	$\text{flip}(0,2)$
$\mathbf{p}_{\text{RBlock}}$	3	Right block position	$\text{flip}(1)$
$\mathbf{R}_{\text{RBlock}}$	4	Right block orientation quaternion	$\text{flip}(1,3)$
$\mathbf{v}_{\text{RBlock}}$	3	Right block linear velocity	$\text{flip}(1)$
$\boldsymbol{\omega}_{\text{RBlock}}$	3	Right block angular velocity	$\text{flip}(0,2)$
Total	26		

Table S26. Extrinsic states and symmetry operators of *Grasp and Place*

Symbol	Dimension	Description	F_{extr}
$\mathbf{p}_{\text{Block}}$	3	Block position	$\text{flip}(1)$
$\mathbf{R}_{\text{Block}}$	4	Block orientation quaternion	$\text{flip}(1,3)$
$\mathbf{v}_{\text{Block}}$	3	Block linear velocity	$\text{flip}(1)$
$\boldsymbol{\omega}_{\text{Block}}$	3	Block angular velocity	$\text{flip}(0,2)$
$\mathbf{p}_{\text{Cup}}$	3	Cup position	$\text{flip}(1)$
$\mathbf{R}_{\text{Cup}}$	4	Cup orientation quaternion	$\text{flip}(1,3)$
$\mathbf{v}_{\text{Cup}}$	3	Cup linear velocity	$\text{flip}(1)$
$\boldsymbol{\omega}_{\text{Cup}}$	3	Cup angular velocity	$\text{flip}(0,2)$
Total	26		

Table S27. Extrinsic states and symmetry operators of *Scissors*

Symbol	Dimension	Description	F_{extr}
$\mathbf{p}_{\text{Scissors}}$	3	Scissors position	$\text{flip}(1)$
$\mathbf{R}_{\text{Scissors}}$	4	Scissors orientation quaternion	$\text{flip}(1,3)$
$\mathbf{v}_{\text{Scissors}}$	3	Scissors linear velocity	$\text{flip}(1)$
$\boldsymbol{\omega}_{\text{Scissors}}$	3	Scissors angular velocity	$\text{flip}(0,2)$
$\mathbf{p}_{\text{LHandle}}$	3	Left handle position	$\text{flip}(1)$
$\mathbf{R}_{\text{RHandle}}$	3	Right handle position	$\text{flip}(1)$
Total	16		

Table S28. Extrinsic states and symmetry operators of *Pen*

Symbol	Dimension	Description	F_{extr}
$\mathbf{p}_{\text{Pen}}$	3	Pen position	$\text{flip}(1)$
$\mathbf{R}_{\text{Pen}}$	4	Pen orientation quaternion	$\text{flip}(1, 3)$
$\mathbf{v}_{\text{Pen}}$	3	Pen linear velocity	$\text{flip}(1)$
$\boldsymbol{\omega}_{\text{Pen}}$	3	Pen angular velocity	$\text{flip}(0, 2)$
$\mathbf{p}_{\text{Grip}}$	3	Pen grip position	$\text{flip}(1)$
$\mathbf{R}_{\text{Cap}}$	3	Cap position	$\text{flip}(1)$
Total	16		

Table S29. Extrinsic states and symmetry operators of *Catch Underarm* and *Catch Abreast*

Symbol	Dimension	Description	F_{extr}
$\mathbf{p}_{\text{Ball}}$	3	Ball position	$\text{flip}(1)$
$\mathbf{R}_{\text{Ball}}$	4	Ball orientation quaternion	$\text{flip}(1, 3)$
$\mathbf{v}_{\text{Ball}}$	3	Ball linear velocity	$\text{flip}(1)$
$\boldsymbol{\omega}_{\text{Ball}}$	3	Ball angular velocity	$\text{flip}(0, 2)$
$\mathbf{p}_{\text{Goal}}$	3	Goal position	$\text{flip}(1)$
$\mathbf{R}_{\text{Goal}}$	4	Goal orientation quaternion	$\text{flip}(1, 3)$
$\hat{\mathbf{R}}_{\text{Goal}}$	4	Goal orientation quaternion in ball frame	$\text{flip}(1, 3)$
Total	24		

Table S30. Hyperparameters in training of asymmetric tasks

Parameter	Value
Actor hidden layers	[512, 256, 128]
Critic hidden layer	[512, 256, 128]
Activation	ELU
Clip range	0.2
Discount factor	0.96
GAE discount factor	0.95
Desired KL divergence	0.016
Learning rate	3×10^{-4}
Number of Environments	2048
Steps per iteration	8
Training iterations	6000
